# Bayesian back-calculation and nowcasting for line list data during the COVID-19 pandemic

**DOI:** 10.1371/journal.pcbi.1009210

**Published:** 2021-07-12

**Authors:** Tenglong Li, Laura F. White

**Affiliations:** Department of Biostatistics, School of Public Health, Boston University, Boston, Massachusetts, United States of America; University of Notre Dame, UNITED STATES

## Abstract

Surveillance is critical to mounting an appropriate and effective response to pandemics. However, aggregated case report data suffers from reporting delays and can lead to misleading inferences. Different from aggregated case report data, line list data is a table contains individual features such as dates of symptom onset and reporting for each reported case and a good source for modeling delays. Current methods for modeling reporting delays are not particularly appropriate for line list data, which typically has missing symptom onset dates that are non-ignorable for modeling reporting delays. In this paper, we develop a Bayesian approach that dynamically integrates imputation and estimation for line list data. Specifically, this Bayesian approach can accurately estimate the epidemic curve and instantaneous reproduction numbers, even with most symptom onset dates missing. The Bayesian approach is also robust to deviations from model assumptions, such as changes in the reporting delay distribution or incorrect specification of the maximum reporting delay. We apply the Bayesian approach to COVID-19 line list data in Massachusetts and find the reproduction number estimates correspond more closely to the control measures than the estimates based on the reported curve.

## Author summary

Interventions meant to control infectious diseases are often determined and judged using surveillance data on the number of new cases of disease. In many diseases, there are substantial delays between the time when an individual is infected or shows symptoms and when the case is actually reported to a public health authority, such as the CDC. This reported data often collects information on symptom onset dates for some individuals. In this paper, we describe a method that imputes missing onset dates for all individuals and recreates the history of the disease progression in a population according to symptom onset dates, which are the best observable proxy available for infection dates. Our method also estimates the instantaneous reproduction number and is robust to many deviations from the assumptions of the model. We show, using a COVID-19 dataset from Massachusetts that our method accurately follows the implementation of control measures in the state.

This is a *PLOS Computational Biology* Methods paper.

## Introduction

Surveillance plays a pivotal role in controlling the COVID-19 pandemic and has been used to provide guidance for government responses to the pandemic [1, 2]. A prerequisite for effective surveillance is to have daily case counts that are ideally defined based on infection dates (called the incidence curve) or, at a minimum, symptom onset dates (called the epidemic curve), which are biologically meaningful [3–5]. However what is most frequently recorded are case reporting dates, which tend to be either the date when an infected individual was tested, tested positive, or reported to public health authorities. The processes that impact the timing of case reporting date, namely obtaining and reporting test results, vary due to a large number of factors, including individual healthcare seeking behaviors, testing availability, or other factors that are not related to disease pathogenesis [6, 7]. This means that the reported curve (daily counts based on case reporting dates) have artificial noise that blurs the underlying epidemiological signal best described by infection dates, or secondarily by symptom onset dates [8, 9]. It also means that it is challenging to obtain timely estimates of the reproduction number as the most recently reported cases represent infection events that occurred some time in the past, which can completely distort the underlying epidemic curve [10]. As these reported curves are often used to estimate reproduction numbers for surveillance and determining the efficacy of interventions, it is important that these cases are reported as close to the actual infection dates as possible [11, 12].

Infection dates are the most epidemiologically meaningful dates as they directly inform infection events and the reproduction numbers. However, obtaining infection dates is very challenging because infection events are not directly observable [12]. This is especially the case for COVID-19 due to significant pre-symptomatic transmission [13]. Typically, infection dates for all cases can only be obtained based on a strong parametric assumption about the distribution of incubation period, which is challenging to estimate [12, 14, 15]. On the other hand, symptom onset dates are more readily observed and in many settings captured for a subset of cases [3]. While symptom onset dates are not as helpful as infection dates, they are still linked to the epidemiology of infectious disease and are typically more proximate to infection events than case reporting dates [3, 16]. This makes the epidemic curve more informative than the reported curve for estimating reproduction numbers [17]. In practice, the major barrier for getting the epidemic curve is that symptom onset dates are still missing for many cases. This makes imputation of reporting delays, which are defined as the lags between symptom onset dates and case reporting dates for individual cases [8–10], a prerequisite for estimating the epidemic curve. The missing reporting delays are due to the ways that cases are reported during the current COVID-19 pandemic. For example, some missing reporting delays are simply due to delays or errors in reporting system as the cases are either not reported yet or their symptom onset dates are missing. However, some other cases have missing reporting delays because they either haven’t been tested yet (asymptomatic cases, test capacity etc.) or haven’t shown symptoms yet (pre-symptomatic cases). In this paper, we focus on addressing missing reporting delays in line list data which refers to a table that stores individual attributes such as dates of symptom onset, reporting or death for each reported case, i.e., each row represented a reported case. In particular, we assume in line list data all cases have known case reporting dates but some of them have missing symptom onset dates.

**Fig 1:**
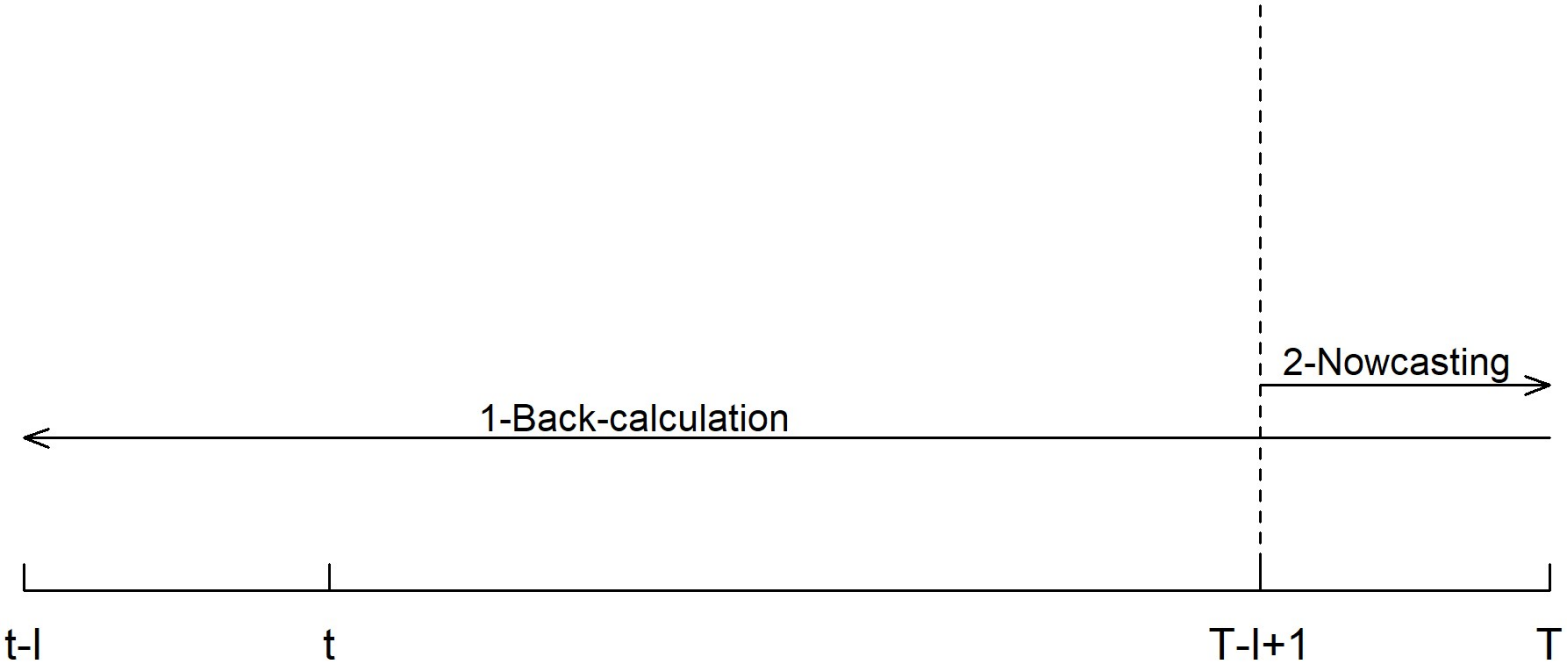
Illustration of back-calculation and nowcasting. Assuming *t* and *T* are the first and last reporting day in a line-list data, one needs to first back-calculate the daily case counts that cover the period from day *t* − *l* to day *T* based on reporting delays, where *l* is the maximum reporting delay. The next step is nowcasting, which is to upscale the back-calculated counts for the period from day *T* − *l* + 1 to day *T*.

Based on observed and imputed reporting delays, there are two steps to recover the epidemic curve from the reported curve. The first step is back-calculation which requires one to back-calculate symptom onset date based on case reporting date for each case [3]. Therefore, the epidemic curve is estimated by the daily case counts based on symptom onset dates rather than case reporting dates. The second step is nowcasting, which is needed because of the reported curve is right truncated, i.e., any case that is reported after the final reporting date (but potentially has symptom onset before the final reporting date) is unavailable for analysis [5]. The consequence of this right truncation issue is that the back-calculated counts of cases that show symptoms on days close to the final reporting date are likely incomplete as some of those cases are actually reported after the final reporting date and unavailable for back-calculation [17]. Hence, nowcasting is the task of modeling and appropriately increasing those case counts. The idea of back-calculation and nowcasting is illustrated by Fig 1.

Most previous work estimates the epidemic curve either by the one-step approach where one models the reporting delay distribution and/or case counts directly [5, 18], or by the two-step approach where one imputes missing reporting delays first (the imputation step) and then recovers the epidemic curve based on the imputed values (the estimation step) [3, 19, 20]. The reporting delay distribution is usually modeled based on the *reporting triangle*, a summary of the empirical distributions of reporting delays based on symptom onset dates [5, 18, 21]. Since the *reporting triangle* does not take missing reporting delays into account, the one-step approach is based on observed reporting delays only and typically is assumed to be time invariant. With such limitations, the two-step approach is generally preferred for a line list data where the missing reporting delays are non-ignorable. In this approach, the imputation step usually assumes symptom onset dates are missing at random conditional on case reporting dates and other available covariates in a line list data [3]. Usually, the imputed reporting delays in the two-step approach are not dynamically updated by the model of the reporting delay distribution, and they may be biased and have large variance. More importantly, making inference about the estimated epidemic curve would be difficult for the two-step approach since the variance associated with the imputation step is not taken into account by the estimation step, if no appropriate multiple imputation step involved.

In this paper, we develop a Bayesian framework that dynamically integrates the imputation step and the estimation step. Our Bayesian framework has five components: (1) inference of the reporting delay distribution based on case reporting dates; (2) imputation of missing reporting delays; (3) back-calculation; (4) nowcasting; and (5) reproduction number estimation using the *EpiEstim* method [22, 23]. The Bayesian framework is simple to implement and suitable for estimating the epidemic curve. We demonstrate the robustness of our framework by simulating an epidemic wave similar to the first COVID-19 outbreak under various conditions, such as changes in reporting delay distribution, violation of model assumptions, and incomplete surveillance data. We also demonstrate that the 95% Bayesian credible intervals of the epidemic curve and reproduction number estimates have good coverage rate even under moderately undesirable conditions and therefore can lead to reliable inferences. We apply this Bayesian method to COVID-19 data in Massachusetts and show that our estimates of the epidemic curve and the reproduction numbers are consistent with the COVID-19 dynamics in Massachusetts.

## Materials and methods

### Imputation of the missing reporting delays

For a line list data, we denote individual case reporting date and symptom onset date as *r*_*i*_ and *t*_*i*_, respectively, for individuals *i* = 1, …, *n*. Therefore, an individual reporting delay is defined as *d*_*i*_ = *r*_*i*_ − *t*_*i*_ and *d*_*i*_ should be non-negative integers. We assume *d*_*i*_ ∈ [0, *l*] for the missing *d*_*i*_. Moreover, we assume reporting starts from day 1 and ends at day *T* in the line list data. We use *t* to denote dates and *t* could be a negative integer. We use *n*_*t*_ to denote the case counts based on the reported curve on day t. The maximum delay *l* can be decided based on the observed reporting delays as well as prior knowledge about the reporting system. The entire reporting period (from day 1 to day *T* in the line list data) can be thought of as the composition of consecutive small reporting periods, such that the reporting delay distribution is stable during each small reporting period. For example, for COVID-19 line list data we can define each week as the small reporting period under the assumption that the reporting delay distribution is unlikely to change sharply within each week. Then, we define *X*_1_ as the *n* × *p* matrix containing the indicators of the small reporting periods and *X*_2_ as the indicator of whether a case is reported on weekends, assuming there are *p* small reporting periods in total (for instance *p* is the number of weeks in the study period). The reporting delay distribution is then modeled for a single spatial region based on case reporting dates:

$$\begin{array}{c} d∼\text{NB}\left(\mu ,r,l\right),\mu =e^{X_{1}\beta +X_{2}\gamma } \end{array}$$ (1)

where *r* and *μ* are the size (dispersion) and mean parameters for negative binomial distribution. *l* represents the upper bound for the above truncated negative binomial distribution.

Sometimes a reporting system improves over time and the reporting delays are significantly shortened after a specific date *t*_*c*_. In this case, Eq (1) is modified as:

$$\begin{array}{c} d∼\text{NB}\left(\mu ,r_{1}1_{t<t_{c}}+r_{2}1_{t\geq t_{c}},l\right),\mu =e^{X_{1}\beta +X_{2}\gamma } \end{array}$$ (2)

where **1**_*A*_ is the indicator of whether the condition *A* is met. In this formulation, Eq (2) has two dispersion parameters: *r*_1_ corresponds to dates prior to *t*_*c*_ and *r*_2_ corresponds to dates equal or later than *t*_*c*_.

### Bayesian inference

Based on Eq (1), the posterior distribution for imputing the missing reporting delays (and thus the missing symptom onset dates) is:

$$\begin{array}{c} f\left(\beta ,r,d^{\text{miss}}|d^{\text{obs}},X_{1},X_{2},l\right)\propto f\left(\beta \right)f\left(\gamma \right)f\left(r\right)f\left(d^{\text{miss}}\right)f\left(d^{\text{obs}}|\beta ,\gamma ,r,d^{\text{miss}},X_{1},X_{2},l\right) \end{array}$$ (3)

where *d*^miss^ represents all the missing *d*_*i*_ and *d*^obs^ represents all the observed *d*_*i*_. Using uninformative priors for *β*, *γ*, *r*, and *d*^miss^, imputation of *d*^miss^ is done via the following Gibbs sampler:

1. sample from *f*(*d*^miss^|*β*, *γ*, *r*, *X*_1_, *X*_2_, *l*)
2. sample from *f*(*β*|*γ*, *r*, *d*^miss^, *β*, *d*^obs^, *X*_1_, *X*_2_, *l*)
3. sample from *f*(*γ*|*r*, *d*^miss^, *β*, *d*^obs^, *X*_1_, *X*_2_, *l*)
4. sample from *f*(*r*|*d*^miss^, *β*, *γ*, *d*^obs^, *X*_1_, *X*_2_, *l*)

where *f*(*d*^miss^|*β*, *γ*, *r*, *X*_1_, *X*_2_, *l*) is a truncated negative binomial distribution whose upper bound is *l*. The above posterior distribution and Gibbs sampler are similarly defined for Eq (2).

To take the impact of testing practice into account, one needs to preprocess the reported curve before using our model. Specifically, the adjusted reported case count ${\tilde{n}}_{t}$, which should be used to adjust for the impact of testing practice, is defined as the ratio between the raw case count *n*_*t*_ and the reporting fraction *q*_*t*_. Since symptom onset dates are the target of imputation, it is impossible to build a model conditional on them. By defining the small reporting periods and modeling the reporting delay distribution for each of these periods, we aim to estimate the reporting delay distribution within each of these periods and thus collectively approximate the underlying reporting delay distribution defined by symptom onset dates. Intrinsically, our approach is data mining rather than statistical modeling of the reporting delay distribution.

### Estimation of the epidemic curve and instantaneous reproduction numbers

Back-calculation is straightforward given the imputed *d*^miss^ and *d*^obs^. The back-calculated counts ${\hat{N}}_{t}$, i.e., the case counts based on symptom onset dates in a line list data, is computed as:

$$\begin{array}{c} {\hat{N}}_{t}=\sum_{i=0}^{n}1_{r_{i}-d_{i}=t},t=-l+1,\ldots ,T. \end{array}$$ (4)

where **1**_*r*_*i*_ − *d*_*i*_ = *t*_ is the indicator of whether the *i*^*th*^ case showed symptoms on day *t*. Assuming the line list data includes all symptomatic cases, we can take ${\hat{N}}_{t}$ as the estimate of *N*_*t*_, the true number of cases who showed symptoms on day *t*, up to day *t* = *T* − *l*. Due to right-truncation ${\hat{N}}_{t}$ likely underestimates *N*_*t*_ for *t* = *T* − *l* + 1, …, *T*. To address this, we define the epidemic curve estimate ${\tilde{N}}_{t}$ (for *t* = *T* − *l* + 1, …, *T*) as the sum of the back-calculated counts ${\hat{N}}_{t}$ and the not-yet-reported counts *s*_*t*_ which should be drawn from the following negative binomial distribution [24]:

$$\begin{array}{cc} & s_{t}∼\text{NB}\left({\hat{N}}_{t},\hat{P}\left(d\leq T-t\right)\right),t=T-l+1,\ldots ,T. \end{array}$$ (5)

$$\begin{array}{cc} & \hat{P}\left(d\leq T-t\right)=\frac{\sum_{i=1}^{n}1_{r_{i}-d_{i}\geq t_{c}}\cdot 1_{d_{i}\leq T-t}}{\sum_{i=1}^{n}1_{r_{i}-d_{i}\geq t_{c}}} \end{array}$$ (6)

If *t*_*c*_ is not provided, it implies there is no change in the reporting system and the indicator $1_{r_{i}-d_{i}\geq t_{c}}$ is always 1. In this case, $\hat{P}\left(d\leq T-t\right)$ is the empirical cumulative density function of the line list data. To summarize, the epidemic curve estimates ${\tilde{N}}_{t}$ are calculated as follows for all dates:

$$\begin{array}{c} {\tilde{N}}_{t}=\{\begin{array}{c} {\hat{N}}_{t},\text{for}\;t=-l+1,\ldots ,T-l\\ {\hat{N}}_{t}+s_{t},\text{for}\;t=T-l+1,\ldots ,T \end{array} \end{array}$$ (7)

With the epidemic curve estimates ${\tilde{N}}_{t},t=-l+1,\ldots ,T$, the instantaneous reproduction number estimates ${\hat{R}}_{t}$ can be obtained based on *EpiEstim* [22, 23] with a sliding window size *τ*:

$$\begin{array}{cc} & {\hat{R}}_{t}=\frac{(\sum_{k=t-\tau }^{t}{\tilde{N}}_{k})+1}{(\sum_{k=t-\tau }^{t}\Lambda_{k}\left(p_{j}\right))+0.2} \end{array}$$ (8)

$$\begin{array}{cc} & \Lambda_{k}\left(p_{j}\right)=\sum_{j=1}^{\text{min}(k,s)}{\tilde{N}}_{k-j}p_{j} \end{array}$$ (9)

Note that the above expression of ${\hat{R}}_{t}$ is actually derived as the posterior mean based on the gamma prior with mean and standard deviation both equal to 5 [22]. The serial interval distribution is needed for computing ${\hat{R}}_{t}$: *s* is the maximum length of serial interval and *p*_*j*_ is the probability of a serial interval of *j* days. Since both the epidemic curve estimate ${\tilde{N}}_{t}$ and reproduction number estimates ${\hat{R}}_{t}$ depend on the imputed reporting delays *d*^miss^, ${\tilde{N}}_{t}$ and ${\hat{R}}_{t}$ are computed based on the posterior sample of *d*^miss^ and updated by the Gibbs sampler for imputation, as well. Therefore, the final output of our Bayesian algorithm is a posterior sample of ${\tilde{N}}_{t}$ and ${\hat{R}}_{t}$. Statistical inference based on their Bayesian credible intervals incorporates the uncertainty about *d*^miss^.

### Overview of simulation study

We simulated a local epidemic similar to COVID-19 using a branching process with the parameters based on COVID-19 literature [11, 14, 25, 26]. From this, we created a line list data based on the simulated epidemic wave (see details in S1 Text). By definition, the branching process started from Feb 1, 2020 and cases reported after March 31, 2020 were excluded in the line list data. For simulation scenarios, we vary three factors: data availability, the maximum reporting delay *l* assumption, and changes in the reporting delay distribution. We considered three possibilities regarding data availability: 1) complete data, 2) delayed surveillance initiation, and 3) real time estimation. The first scenario is ideal with the line list data covering the entire epidemic wave. In the second scenario, the line list data is only available after a certain date during the epidemic wave, possibly due to delays in initiating surveillance. In this case, we explored four different starting dates for the line list data to reflect various degrees to which earlier reports were lost and explore the impact of these delays on our approach. Third, we focus on estimation in the midst of the epidemic wave, which means the final reporting date in the line list data is prior to the end of the epidemic wave. We chose two different final reporting dates for the line list data: 1) before the peak of the reported curve, and 2) after the peak of the reported curve.

We also tested the case where we assumed *l* was 20 days for estimation when *l* actually was 25 days. We considered three possible scenarios regarding the changing dynamics of the reporting delay distribution over time. First, the reporting delay distribution remained unchanged and there was no improvement throughout the epidemic wave. The average reporting delay was 9 days in this case. Second, the reporting delay distribution sharply improved to an average of 4 days in the middle of the epidemic wave (*t*_*c*_ = March 1, 2020 based on symptom onset dates). Third, the reporting delay distribution was constantly and gradually improving during the epidemic wave. The average reporting delay gradually decreased from 9 days at the beginning to 4 days at the end of the epidemic wave.

We simulated 1000 line list datasets for each of the 18 different simulation scenarios. On average, the line-list data included about 5000 cases reported over 54 days. We randomly made the symptom onset dates missing for 60% of the cases, a percentage that was consistent with the CDC line list data.

### Line list data of COVID-19 cases in Massachusetts

We apply our method to a CDC line list data for Massachusetts with 85,627 COVID-19 cases reported from Jan 1, 2020 to May 14, 2020. 823 cases were excluded from analysis due to negative reporting delays, which cannot be handled by our model. We expect the exclusion of those cases to have little impact on downstream analyses as those cases are less than 1% of the total cases and they are evenly distributed over the reporting period (the maximum and minimum of the weekly proportions of cases who have negative reporting delays are 2.8% and 0.6% respectively). We excluded 5 cases that were reported before March 4, 2020, as they were discontinuously and sparsely distributed during the period. We set the maximum reporting delay to 60 days, marking 102 cases with longer reporting delays (ranging from 61 days to 117 days) as missing. Based on the data, these 102 reporting delays were clear outliers, potentially due to data entry errors. The final line list data contained 84,799 cases reported from March 4, 2020 to May 14, 2020 with symptom onset dates missing for 61.3% of the cases. Each of the 11 weeks was defined as the small reporting period for model estimation. We also calculated the average daily flow rates based on the daily flow rates of all Massachusetts counties extracted from the *SafeGraph* data [27] for the period between Feb 21, 2020 and May 14, 2020, in order to check whether our reproduction number estimates were consistent with the mobility pattern in Massachusetts. The code and simulation output are available at https://github.com/tenglongli/backandnow. The COVID line list data is available through the CDC and requires access request.

## Results

### Simulation results: Complete line list data and delayed surveillance initiation

To ensure convergence of the Markov Chain Monte Carlo (MCMC) algorithm, the posterior sample was obtained based on 21,000 MCMC iterations with 1000 burn-in iterations for each of the 1000 simulated datasets. We ran Geweke’s convergence diagnostic to check the convergence of MCMC algorithm, and 92% of the estimates (epidemic curve and reproduction numbers) passed the test on average [28]. For all reproduction number estimation, the serial interval was assumed to follow the gamma distribution with the shape equal to 4.29 and the rate equal to 1.18 [14, 29], and the maximum serial interval was assumed to be 14 days. The median and 95% Bayesian credible intervals of the posterior samples of ${\tilde{N}}_{t}$ and ${\hat{R}}_{t}$ were extracted for each simulated dataset. The known epidemic curve and estimated reproduction numbers for each dataset served as the simulation benchmarks. To demonstrate the difference between the epidemic and reported curves, the reported curve and the reproduction number estimates based on it were also obtained for each dataset. The estimates were evaluated by two metrics: 1) the actual coverage rate of the 95% Bayesian credible interval based on 1000 simulated datasets, and 2) the root mean square error (RMSE) calculated as follows:

$$\begin{array}{c} \text{RMSE}=\sqrt{\frac{1}{m}\sum_{j=1}^{m}{\left(x_{j}-y_{j}\right)}^{2}} \end{array}$$ (10)

where *m* is the number of simulated datasets. *x*_*j*_ and *y*_*j*_ are the estimate and benchmark for *j*^*th*^ dataset.

**Fig 2:**
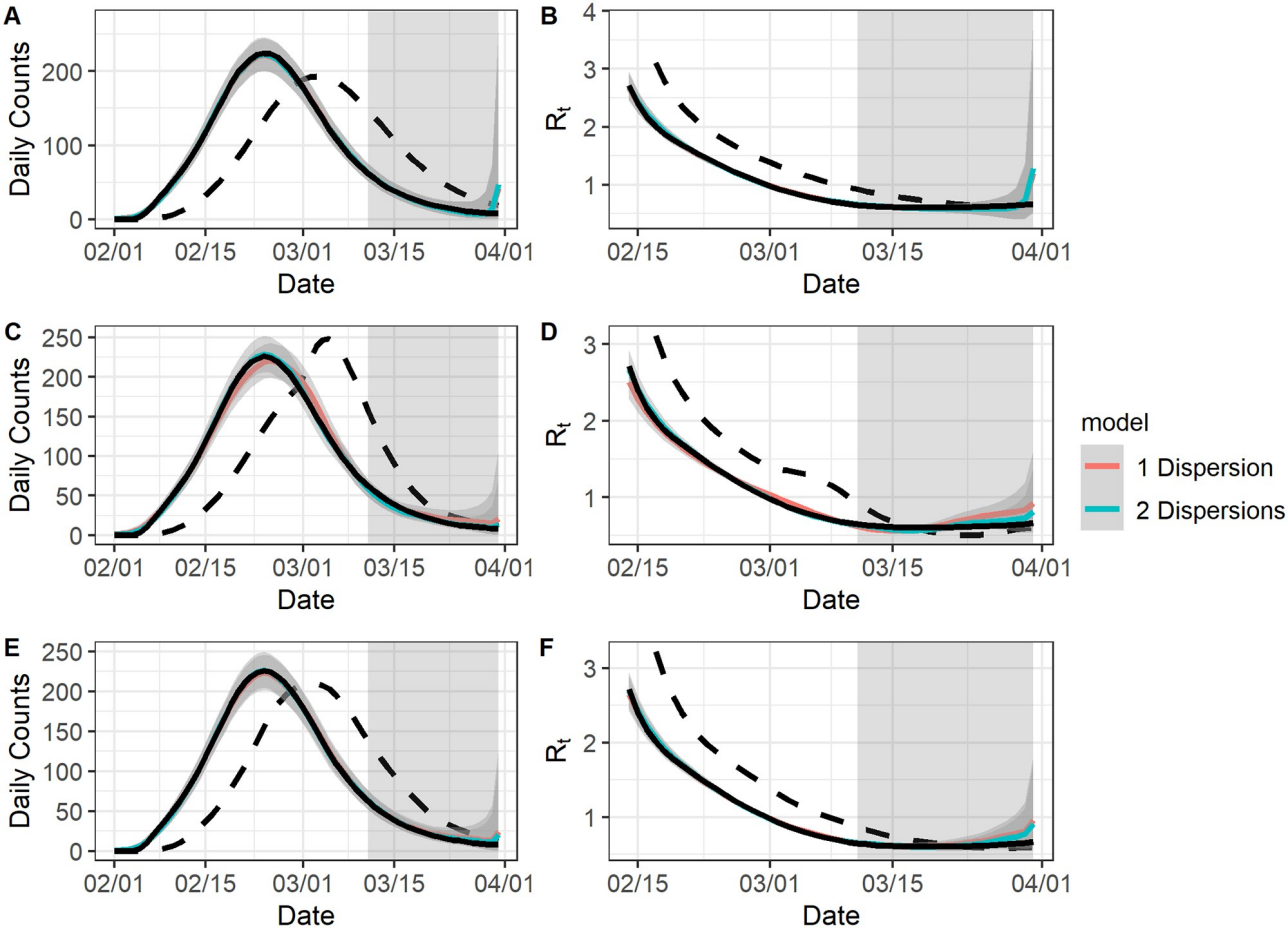
The model fit for complete data. For all graphs: the black solid curve corresponds to estimates based on the known epidemic curves and the black dashed curve corresponds to estimates based on the reported curves. The grey-shaded region superimposed on the curve depicts the 95% Bayesian credible interval and the grey-shade region on the right indicates the region of nowcasting. The colored curves represent different model choices. All values were averaged over 1000 simulated datasets with the correct *l*. A: The epidemic curve estimates if the reporting delay distribution was unchanged. B: The reproduction number estimates if the reporting delay distribution was unchanged. C: The epidemic curve estimates if the reporting delay distribution was sharply improved. D: The reproduction number estimates if the reporting delay distribution was sharply improved. E: The epidemic curve estimates if the reporting delay distribution was gradually improved. F: The reproduction number estimates if the reporting delay distribution was gradually improved.

As expected, the estimated epidemic curve and reproduction numbers were much closer to the simulation benchmark than the reported curve and the corresponding reproduction number estimates. On average, the estimated reproduction numbers based on the epidemic curve were 13 days behind the true reproduction numbers built on the dates of infection, due to incubation periods and the sliding window size (*τ* = 6) we chose for *EpiEstim*. With complete line list data, our model estimated true epidemic curve and the reproduction number well and was not sensitive to the changes in the reporting delay distribution (Fig 2). The estimates were not sensitive to the assumption about the maximum delay *l* across all simulation scenarios. For example, we illustrated the impact of the maximum delay assumption for complete line list data. (S1 and S2 and S3 Figs). Therefore, we only discuss the results obtained under the correct maximum delay assumption in the main text henceforth.

Our estimates under the scenario where the reporting delay distribution improved sharply on March 1, 2020 during the epidemic wave (Fig 2C and 2D) was comparatively worse than other two scenarios (i.e., when the reporting delay distribution was either not improved or gradually improved), as manifested by the underestimation of the reporting delays between Feb 15, 2020 and March 8, 2020 during the epidemic wave. The underestimation was mainly due to the overlap of the two reporting delay distributions for cases reported from March 1, 2020 to March 20, 2020 (most of whom had symptom onsets from Feb 15, 2020 to March 8, 2020). Our model struggled to separately estimate the two distributions during this period because it is built on case reporting dates rather than symptom onset dates. We also used both the Eqs (1) and (2) for imputation and estimation. The two models performed similarly when the reporting delay distribution was unchanged or gradually improved. However, Eq (2) did result in a slightly better fit than Eq (1) when the reporting delay distribution sharply improved, likely due to having two dispersion parameters.

Table 1 lists the coverage rate of 95% Bayesian credible interval and the RMSE for our estimates. The average coverage rate of our epidemic curve estimates was 0.89 when there was an abrupt improvement for the reporting delay distribution and was 0.95 when there was gradual or no change in the reporting delay distribution. The average coverage rates of the reproduction number estimates was slightly lower than the average coverage rates of the epidemic curve estimates in general, likely due to the additional error brought by *EpiEstim* [23]. Compared to Eq (1), Eq (2) had higher coverage rates and RMSE of the epidemic curve estimates when the reporting delay distributions sharply improved on March 1, 2020 (coverage rate: from 0.89 to 0.94; RMSE: from 8.92 to 7.60). The gain of using Eq (2) was even larger for the reproduction number estimates in this case: the coverage rate increased from 0.64 to 0.87 and the RMSE decreased from 0.08 to 0.06. For the other two scenarios, Eq (2) was comparable to Eq (1). Overall, the Bayesian credible interval was tight (indicated by the small RMSE) with the nominal coverage rate (around 0.95) given appropriate model choice, when the line list data was complete for the epidemic wave.

**Table 1:** Performance measures for complete data. The results were averaged over all simulated datasets and dates for both the epidemic curve (Curve) and the reproduction numbers (*R*_*t*_). The results format: coverage rate (RMSE). Model 1 refers to the model in Eq (1) and model 2 refers to the model in Eq (2).

Improvement	Maximum Delay	Model 1		Model 2	
		Curve	R_t_	Curve	R_t_
No	Correct	0.96 (7.34)	0.94 (0.05)	0.96 (7.44)	0.94 (0.06)
	Incorrect	0.95 (7.60)	0.90 (0.06)	0.96 (7.85)	0.90 (0.07)
Sharp	Correct	0.90 (8.76)	0.64 (0.08)	0.95 (7.02)	0.88 (0.05)
	Incorrect	0.88 (9.07)	0.63 (0.08)	0.93 (8.17)	0.85 (0.06)
Gradual	Correct	0.95 (7.28)	0.90 (0.05)	0.96 (6.85)	0.93 (0.05)
	Incorrect	0.95 (7.26)	0.91 (0.05)	0.96 (6.97)	0.92 (0.05)

We also checked the coverage rate and RMSE of the case count estimates on each day based on symptom onset dates, in order to evaluate the performance of our model from a temporal perspective (S4 and S5 Figs). Overall, the coverage rate was negatively correlated with the RMSE, consistent with of our other results. The coverage rate of our estimate was consistently over 0.9 for all the dates, except when the reporting delay distribution did sharply improve during the epidemic wave and Eq (1) was used. We also notice that the credible interval became much wider for the last several days compared to other dates, which was probably due to the fact that the right truncation issue was the worst for those days, i.e., most of the cases that showed symptoms on those days were to be reported after the final reporting date (March 31, 2020) of the line-list data and thus unavailable for analysis.

**Fig 3:**
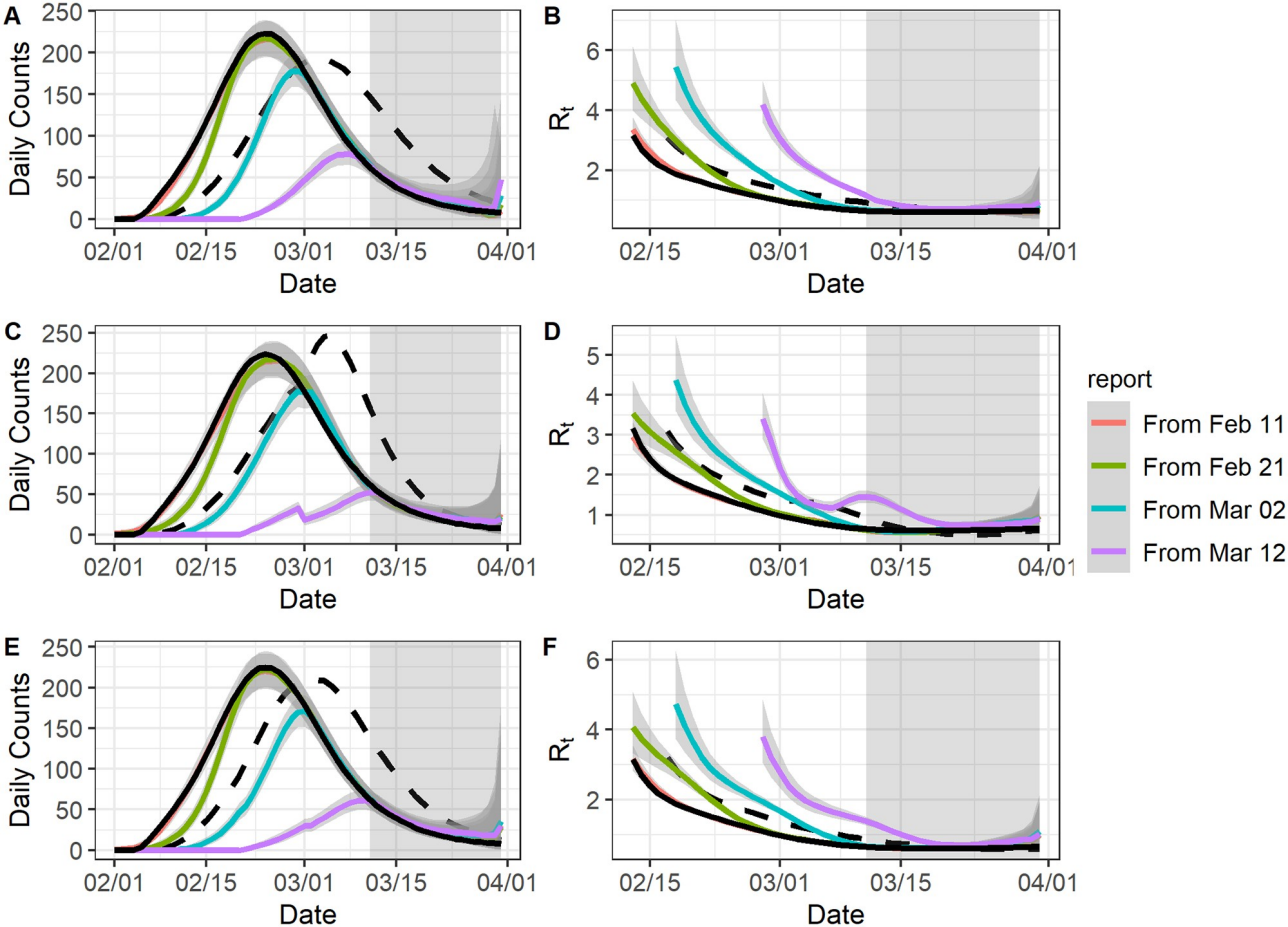
The model fit for data with no early report. For all graphs: the black solid curve corresponds to estimates based on the known epidemic curves and the black dashed curve corresponds to estimates based on the reported curves. The grey-shaded region superimposed on the curve depicts the 95% Bayesian credible interval and the grey-shade region on the right indicates the region of nowcasting. The colored curves represent different starting dates for the line-list data. All values were averaged over 1000 simulated datasets with the correct *l*. A: The epidemic curve estimates if the reporting delay distribution was unchanged. B: The reproduction number estimates if the reporting delay distribution was unchanged. C: The epidemic curve estimates if the reporting delay distribution was sharply improved. D: The reproduction number estimates if the reporting delay distribution was sharply improved. E: The epidemic curve estimates if the reporting delay distribution was gradually improved. F: The reproduction number estimates if the reporting delay distribution was gradually improved.

For delayed surveillance initiation, we assume four different starting dates for the line list data: Feb 11, 2020, Feb 21, 2020, March 2, 2020, and March 12, 2020. To enhance comparability of the results based on the line-list data with different starting dates, we only used the Eq (1) for estimation. In general, we estimate the epidemic curve well from the starting date onward (Fig 3). For reproduction number estimation, the estimates become reliable *τ* + 1 days after the starting date, since *EpiEstim* needs at least *τ* + 1 days’ observations to produce unbiased estimates. For example, if the starting date is Feb 11, 2020 and *τ* = 6 one should expect the epidemic curve and reproduction number estimates to converge to their benchmarks from Feb 11, 2020 and Feb 18, 2020 respectively. In general, estimation accuracy decreases with longer delays (Table 2). For individual daily case counts, the coverage rate (S6 Fig) and the RMSE (S7 Fig) were acceptable after the starting date. We still observe that the estimated epidemic curve and reproduction numbers were far better than the reported curve and its associated reproduction numbers, unless there was a severe loss of early reporting (eg. if the starting date was March 2, 2020 or March 12, 2020).

**Table 2:** Performance measures for data with no early report. The results were averaged over all simulated datasets and dates for both the epidemic curve (Curve) and the reproduction numbers (*R*_*t*_). The line-list data could start on Feb 11, 2020 (Data 1), Feb 21, 2020 (Data 2), March 2, 2020 (Data 3) or March 12, 2020 (Data 4). The results format: coverage rate (RMSE).

Improvement	Maximum Delay	Data 1		Data 2		Data 3		Data 4	
		Curve	R_t_	Curve	R_t_	Curve	R_t_	Curve	R_t_
No	Correct	0.93 (8.03)	0.87 (0.07)	0.73 (14.28)	0.61 (0.30)	0.55 (39.05)	0.43 (0.58)	0.39 (69.76)	0.21 (0.68)
	Incorrect	0.92 (9.11)	0.85 (0.07)	0.72 (14.58)	0.58 (0.28)	0.54 (38.81)	0.43 (0.51)	0.39 (68.46)	0.20 (0.58)
Sharp	Correct	0.91 (8.90)	0.67 (0.08)	0.71 (14.34)	0.37 (0.21)	0.54 (37.74)	0.33 (0.46)	0.38 (70.70)	0.14 (0.53)
	Incorrect	0.89 (9.40)	0.63 (0.08)	0.70 (14.60)	0.38 (0.19)	0.53 (37.34)	0.33 (0.40)	0.38 (70.19)	0.17 (0.44)
Gradual	Correct	0.95 (7.63)	0.89 (0.06)	0.74 (13.72)	0.58 (0.25)	0.55 (41.01)	0.37 (0.53)	0.39 (70.31)	0.16 (0.66)
	Incorrect	0.95 (7.66)	0.90 (0.06)	0.74 (13.65)	0.58 (0.23)	0.55 (40.63)	0.38 (0.47)	0.39 (70.13)	0.17 (0.58)

### Simulation results: Real time estimation

**Fig 4:**
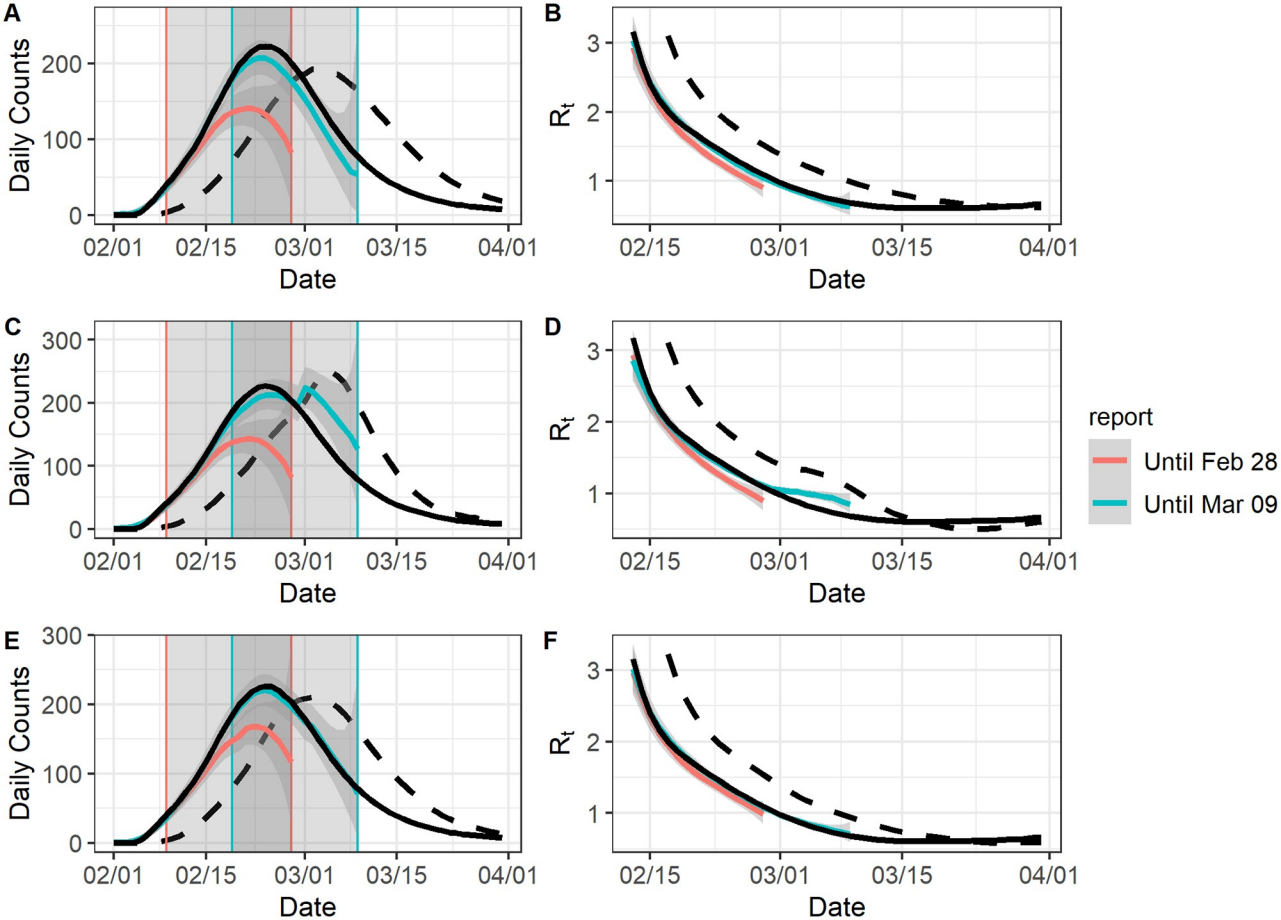
The model fit for an ongoing epidemic wave. For all graphs: the black solid curve corresponds to estimates based on the known epidemic curves and the black dashed curve corresponds to estimates based on the reported curves. The grey-shaded region superimposed on the curve depicts the 95% Bayesian credible interval. The colored curves represent different ending dates for line-list data, and their nowcasting regions are displayed as the gray-shaded areas with boundary lines in their corresponding colors. All values were averaged over 1000 simulated datasets. All values were averaged over 1000 simulated datasets with the correct *l*. A: The epidemic curve estimates if the reporting delay distribution was unchanged. B: The reproduction number estimates if the reporting delay distribution was unchanged. C: The epidemic curve estimates if the reporting delay distribution was sharply improved. D: The reproduction number estimates if the reporting delay distribution was sharply improved. E: The epidemic curve estimates if the reporting delay distribution was gradually improved. F: The reproduction number estimates if the reporting delay distribution was gradually improved.

We chose Feb 28, 2020 (before the peak of the reported curve) or March 9, 2020 (after the peak of the reported curve) as the final reporting dates for the line-list data. As in the previous section, we only used Eq (1) for estimation to ensure comparability of the results. If the final reporting date was Feb 28, 2020, we consistently underestimated the epidemic curve and the reproduction numbers (Fig 4). The average coverage rates were low (epidemic curve: 0.55, reproduction number: 0.40) and the RMSE were large (epidemic curve: 35.94, reproduction number: 0.16). By comparison, the average coverage rates were much higher if the final reporting date was March 9, 2020 (epidemic curve: 0.84, reproduction number: 0.74), and in this case the RMSE were much lower (epidemic curve: 16.50, reproduction numbers: 0.08) (Table 3).

**Table 3:** Performance measures for an ongoing epidemic wave. The results were averaged over all simulated datasets and dates for both the epidemic curve (Curve) and the reproduction numbers (*R*_*t*_). The line-list data could end on Feb 28, 2020 (Data 1) or March 9, 2020 (Data 2). The results format: coverage rate (RMSE).

Improvement	Maximum Delay	Data 1		Data 2	
		Curve	R_t_	Curve	R_t_
No	Correct	0.54 (37.87)	0.33 (0.18)	0.88 (15.54)	0.76 (0.06)
	Incorrect	0.50 (40.72)	0.33 (0.18)	0.82 (17.69)	0.73 (0.07)
Sharp	Correct	0.54 (38.47)	0.32 (0.18)	0.74 (22.07)	0.56 (0.11)
	Incorrect	0.50 (40.41)	0.33 (0.18)	0.71 (22.67)	0.54 (0.11)
Gradual	Correct	0.62 (28.33)	0.53 (0.12)	0.96 (10.31)	0.92 (0.05)
	Incorrect	0.58 (29.82)	0.56 (0.12)	0.95 (10.49)	0.93 (0.05)

Interestingly, our model had the best performance when there was gradual improvement in the reporting delay distribution, especially if the final reporting date was March 9, 2020 for the line list data. In this case, the coverage rates and RMSE of the estimates were very close to those for complete data, and the coverage rates were consistently around 0.9 for all individual daily case counts (S8 and S9 Figs). In the other two scenarios, the coverage rates and RMSE were much worse and very unstable. This is because our model approximated the gradually improving reporting delay distribution well as it was built on the small reporting periods, which could be perceived as smoothing windows and lead to good local estimates. This is also because most part of the epidemic curve and reproduction number estimation was done by nowcasting (Fig 4), which benefits from gradually improved reporting delays. When there was no improvement in the reporting delay distribution, underestimation is worse due to more extreme right truncation. When there was a sharp improvement for the reporting delay distribution, we observed an erratic sudden jump of the daily count estimates, which likely resulted from nowcasted case counts being overweighted as reporting delays tended to be underestimated, a pattern that had been observed for the complete data.

### COVID-19 in Massachusetts

**Fig 5:**
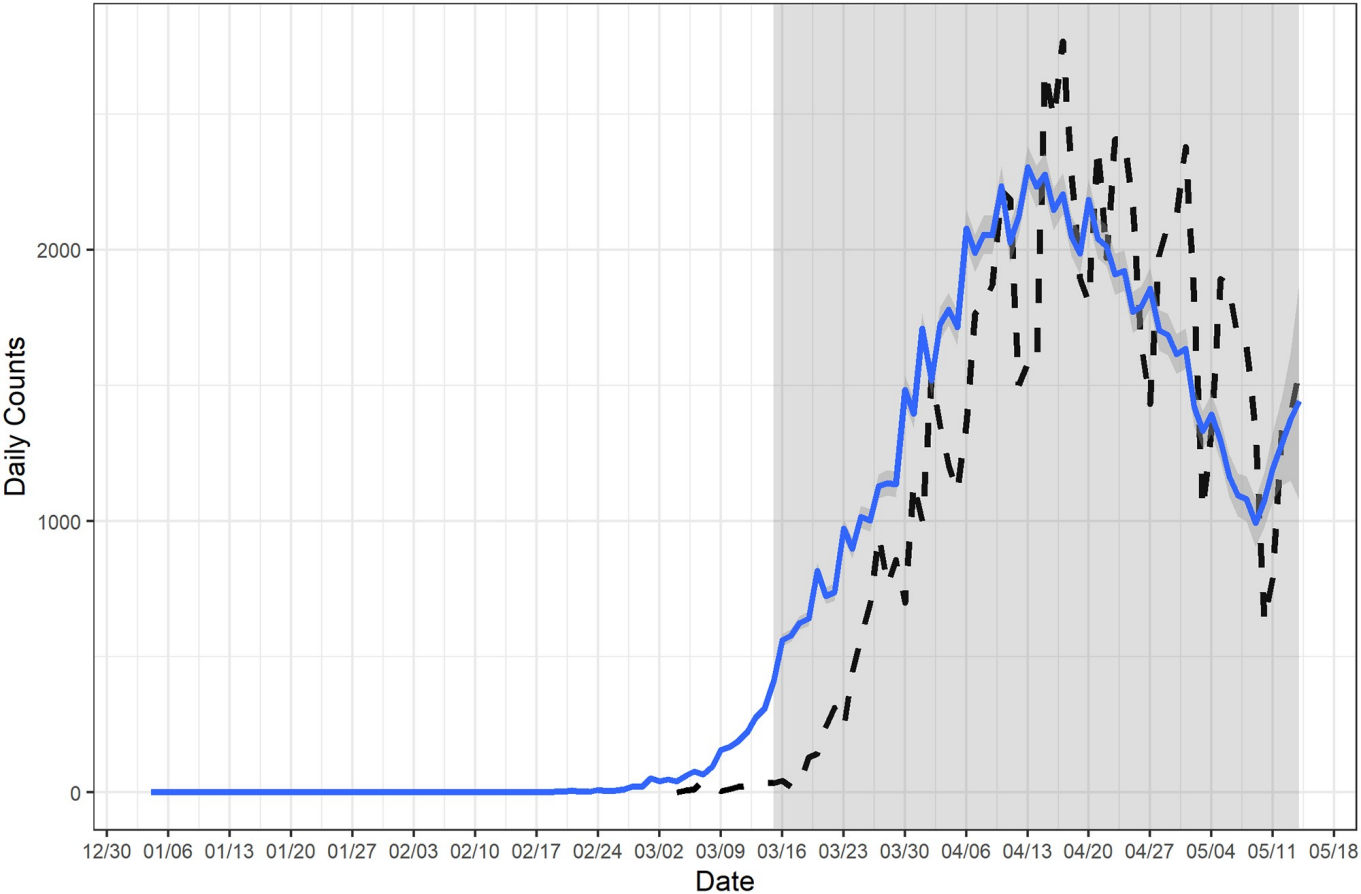
Estimated epidemic curve of COVID-19 in Massachusetts. The estimated epidemic curve was calculated based on weekly smoothing window and *l* = 60. The line-list data started on March 4, 2020 and ended on May 14, 2020. The earliest possible date that a case showed symptoms was February 1, 2020 and nowcasting started from March 16, 2020. The dashed curve represents the reported curve.

**Fig 6:**
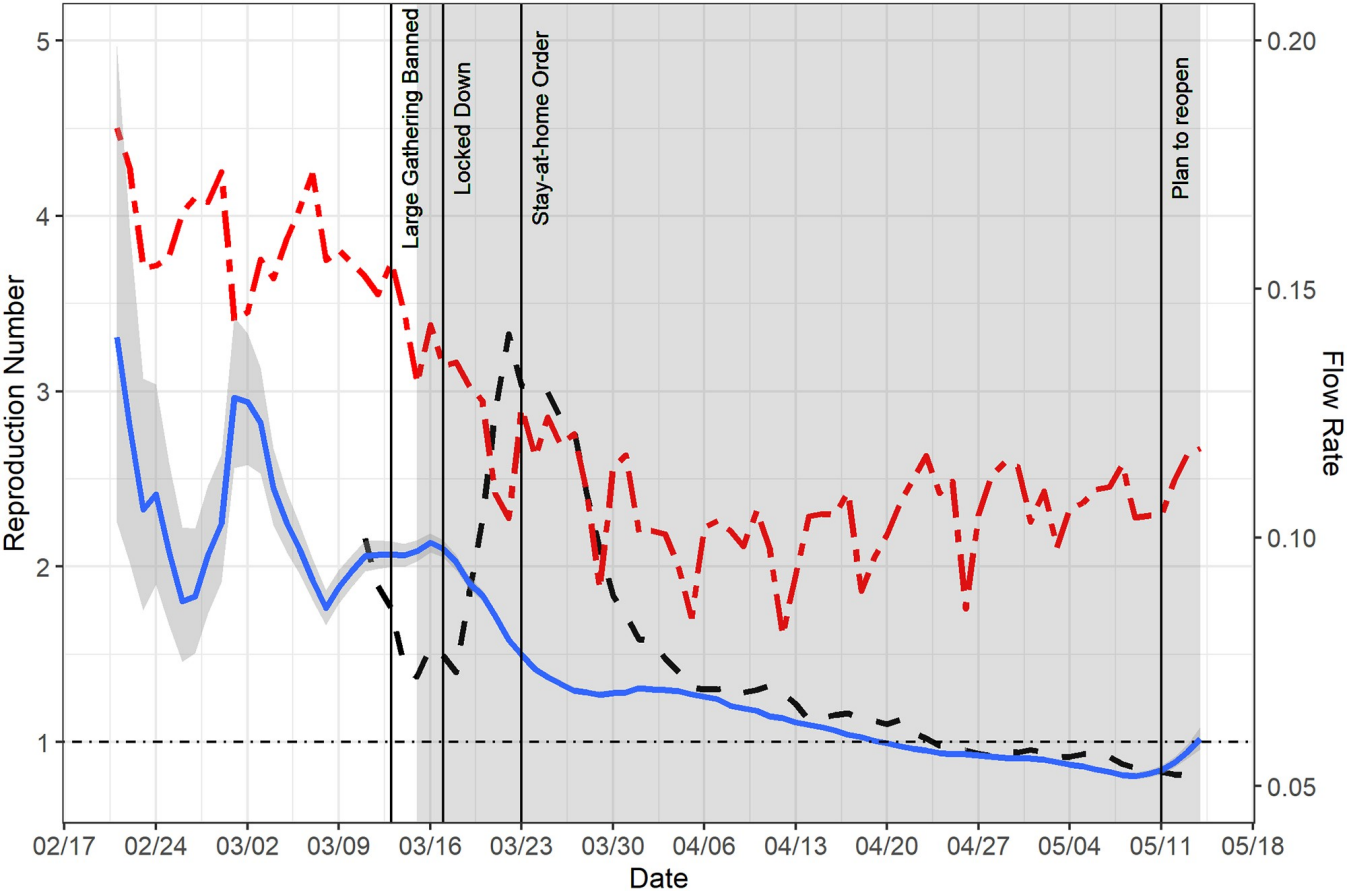
Estimated instantaneous reproduction number of COVID-19 and daily flow rates in Massachusetts. The estimates were calculated based on *EpiEstim* and a posterior sample of epidemic curve estimates (the blue curve). We identify the dates for four key policies: large gathering banned (March 13, 2020), lock-down (March 17, 2020), stay-at-home order (March 23, 2020) and the plan of reopening (May 11, 2020). By comparison, the reproduction number estimates based on the reported curve are described by the dashed black curve. The average daily flow rates over all the counties in Massachusetts was overlaid on the same plot to illustrate the mobility pattern (the red curve).

We estimated the epidemic curve based on the COVID-19 line list data in Massachusetts and compared it with the reported curve (Fig 5). The estimated epidemic curve was much smoother than the reported curve, which indicates that most of the fluctuations in the reported curve were artificial. The estimated epidemic curve suggests that the daily count of COVID-19 cases showing symptoms started to increase in early March in Massachusetts, and the daily count began to decline around mid April with a slight increase around May 10. In addition, we estimated instantaneous reproduction numbers based on the estimated epidemic curve, assuming the distribution of serial interval is Gamma(4.29, 1.18) and *τ* = 6 [14, 17, 29]. We estimated that the instantaneous reproduction numbers were above 2 during the initial stage of the outbreak and the reproduction number started to drop around mid March (Fig 6). The estimated reproduction number dropped below 1 since mid April but rose again around May 11. The trajectory of the estimated reproduction numbers suggests that the reproduction number would likely exceed the critical threshold of 1 after May 14.

To examine the credibility of our reproduction number estimates, we marked the dates of the non-pharmaceutical interventions (NPI) implemented by the state of Massachusetts (Fig 6). Specifically, the state of Massachusetts banned large gathering on March 13 and was locked down from March 17. The governor issued the stay-at-home order on March 23. In addition, we utilized the *SafeGraph* data [27] to illustrate the daily mobility pattern in the state of Massachusetts during the same period. The daily mobility is quantified by the daily flow rates which are ratios of the number of residents moving out and the total number of residents for all counties in Massachusetts. The average daily flow rate (over all the counties) is depicted in Fig 6 as well. We have three key observations: First, the lock-down and stay-at-home order likely reduced the mobility for about 30–35% (from 0.16 to 0.11), which is consistent with literature that suggests lock-down and stay-at-home order are effective in reducing the mobility [30, 31]. Second, our estimated instantaneous reproduction numbers are closely aligned with the daily mobility pattern, suggesting that the lock-down and stay-at-home order are effective for controlling the COVID-19 outbreak in Massachusetts. Previous research also found the NPIs, including lock-down and stay-at-home order, were effective for containing the COVID-19 outbreak and reducing the public healthcare burden in the state of Massachusetts [32], in the United States [33] and worldwide [34, 35]. Third, we observed the daily mobility was slowly increasing from May 2020, and this could explain why the reproduction number estimates rose again around May 11, at which point the reopening plan was unveiled for Massachusetts. Most importantly, we found that the reproduction number estimates based on the estimated epidemic curve corresponded much more closely to the NPIs and the daily mobility pattern in Massachusetts than those based on the reported curve, which demonstrates the necessity for adjusting the raw reported curve using our Bayesian method.

We caution readers about the limitations of our analysis. First, we only back-calculated the symptom onset dates from the case reporting dates in this example and thus compared the instantaneous reproduction numbers based on the symptom onset dates with the timing of interventions. Ideally, as discussed earlier, one should further back-calculate the infection dates and estimated the incidence curve, which is most appropriate for calculating the reproduction numbers. For example, if we subtract the mean incubation period (5 days) [25, 26] from all symptom onset dates, the reproduction number would start to decrease from March 12 and start to increase again from May 6. The resultant reproduction numbers based on such incidence curve would be an even closer match with the daily mobility pattern, compared with the ones based on the epidemic curve. However, since the Massachusetts line list data didn’t have individual incubation periods or dates of infection, we decided to focus on individual symptom onset and case reporting dates and build the Bayesian framework thereof. We note that back-calculation to symptom onset dates, in this case (i.e., when the dates of infection are unavailable for all reported cases), is more robust and convenient than back-projection to dates of infection, which heavily relies on parametric assumption of incubation periods based on external data and likely introduces considerable noise brought by additional imputations. Second, the impact of testing practice was not taken into account in our analysis, as no data on testing practice in Massachusetts is available. Testing practice has a profound impact on reproduction number estimation and its impact has been extensively studied for COVID-19 surveillance [36]. As a result, our reproduction number estimates may subject to bias due to fluctuations in the reporting fraction. Third, our results may indicate there was a causal relationship between the NPI and the drop in reproduction numbers, however such relationship may not be warranted without a thorough study about the potential confounders. For example, if people had changed their behaviors out of their own consciousness or under the guidance of others (like elder people living in nursing homes) before the implementation of the NPI, the effect of the NPI may not be as significant as we thought it would be.

## Discussion

Reproduction numbers are urgently needed for monitoring the progression of the COVID-19 pandemic, and they should be estimated based on reliable epidemic curve estimates, rather than the reported curve, to ensure minimal loss of the epidemiological signal. We introduce a Bayesian approach to estimate the epidemic curve and instantaneous reproduction numbers from line list data. This approach has two unique advantages over other similar approaches. First, it is built on line list data which contains individual reporting delays that allow the estimation of the reporting delay distribution to be data-oriented and time-dependent. Second, it integrates the tasks of estimation of the reporting delay distribution, imputation of the reporting delay as well as estimation of the epidemic curve and reproduction numbers into one Bayesian framework, making those three tasks interdependent. As a result, our approach more accurately estimates uncertainty and is more efficient than other approaches that perform the three tasks independently. The results suggest the Bayesian approach is robust to unfavorable changes in data availability and misspecification of the reporting delay or the maximum delay assumption. Under typical assumptions, the Bayesian approach produces accurate estimates (low RMSE) and reliable inference (high coverage rate).

Unsurprisingly, the model performance does rely on data availability, and it will be inadequate based on insufficient data. For a single epidemic wave, our model estimates both the epidemic curve and reproduction numbers well if line list data is available for the whole epidemic wave, though one should be cautious about the model choice if the reporting system has significantly improved over time. If there are severe delays in initiating surveillance, our model will likely underestimate the case counts of the days prior to the starting date of surveillance, and the *R*_*t*_ estimates will eventually converge at a rate consistent with the serial interval. If estimation is performed in the midst of an outbreak, the Bayesian approach will underestimate the epidemic curve before the peak of the reported curve but performs substantially better after the peak. This suggests that, in the case of single epidemic wave, we need to wait until the peak of the reported curve has passed to ensure there is sufficient data for estimating the reproduction number using this approach. We stress that, if a line list data contains multiple epidemic waves, the Bayesian estimates are at least accurate for all except the last epidemic wave. To safely estimate the last epidemic wave, one still needs to wait until the majority of its cases are reported.

Our model could be sensitive to sharp changes in the reporting delay distribution. If the reporting delay distribution remains unchanged or changes gradually, our model generally performs well. However, if there is a sharp improvement for the reporting delay distribution, using Eq (1) will generate inaccurate estimates during the period when the two underlying reporting delay distributions overlap, resulting in underestimation of reporting delays. In this case, it would be beneficial to use Eq (2) to fit the reporting delay distribution instead. In general, we recommend using Eq (2) for the reporting delay distributions with sharp changes and Eq (1) for those without sharp changes. We also note that our model may not be adequate for handling sharp changes that are due to quality controls in reporting systems, and in this case there are better alternatives, such as the discrete time hazard models [18, 19].

Our model generates a posterior sample of instantaneous reproduction number estimates, based on the epidemic curve estimates. We use *EpiEstim* to compute instantaneous reproduction numbers, conditional on the maximum length and distribution of serial interval. We choose *EpiEstim* because it is more appropriate for real-time analysis and tracking of temporal changes (such as impact of a policy), compared to other alternatives [17]. We recommend using an integrated approach that includes both inference of the reporting delays and estimation of reproduction numbers, to incorporate all sources of uncertainty in modeling, since we are better able to estimate variability due to estimation from this multistage process. We note a few limitations of our approach that are inherited from the *EpiEstim* estimator. First, the maximum length of serial interval *s* and the sliding window size *τ* are subjective choices [23]. Second, it is possible to have negative serial intervals for COVID-19 which is currently not allowed by *EpiEstim* [37]. We note that the reproduction number estimates are potentially biased if the serial interval could be negative. Third, it is most accurate to estimate reproduction numbers from the incidence curve rather than the epidemic curve for *EpiEstim* [23, 38]. However, infection events would be very hard, if not impossible, to observe for the current pandemic and thus a distribution of incubation period is needed for obtaining the infection dates [12, 14], which is typically done via an additional back-projection step based on the estimated epidemic curve and not the focus of this paper. Fourth, reproduction number estimates will be less trustworthy if the fraction of infection observed is not constant over time [3, 22, 39]. For COVID-19, this is likely the case considering the evolution of testing and the significant proportion of asymptomatic transmission [36], requiring further adjustment of the data.

It is worthy emphasizing that we only focus on one part in the workflow for processing the reported curve and/or line list data, i.e., estimating the epidemic curve and the instantaneous reproduction numbers thereof. The whole workflow should also include one preprocessing step where the data is adjusted for testing practice (i.e., fluctuations in the reporting fractions) and one postprocessing step where the dates of infection are further back-calculated based on the dates of symptom onset. In this paper, we take a data-oriented approach, i.e., what we can achieve based on available line list data and the line list data only. However, it’s important to acknowledge that the preprocessing step is much needed as the impact of testing practice on reproduction number estimation is profound [36]. As outlined in the method section, one should estimate the reporting fractions based on data specifically on testing practice or other proxy data such as the proportions of hospitalization among COVID-19 cases [3], and compute the adjusted reported case counts ${\tilde{n}}_{t}$ under our framework. Unfortunately, data on testing practice is rarely available during the current pandemic, which undermines the validity of most reproduction number estimates. Moreover, the postprocessing step is usually necessary as the incidence curve is preferred for estimating the reproduction numbers. Ideally, a line list data should have additional variable containing the dates of infection at the individual level, but this is hardly the case for the current COVID-19 pandemic. Consequently, the postprocessing step for obtaining the dates of infection is typically built on a parametric distribution of incubation periods, which does not involve line list data and thus is beyond the scope of this paper.

Empirically, there are some important issues to consider in properly implementing our method. First, our model is region-specific, i.e., one need to fit our model to line list data of a single region to avoid systematic differences between regions. The region is defined such that each region is deemed to have its own reporting system (and thus its unique reporting delay distribution). For example, if the reporting system differs at the county level, we should use line list data of each county (rather than each state) for our model. Second, the reporting period in our model needs to be carefully and properly defined, as our model is essentially a moving-window smoothing method. As with most other moving-window smoothing methods, the model performance depends on the moving-window size, which in our case is the reporting period size [17]. The moving-window size is known for its pivotal role in the bias-variance trade-off and thus should be neither too small nor too large for estimating the reporting delay distribution [5]. Third, our model cannot handle negative reporting delays which are possible for the current COVID-19 pandemic due to contact tracing, though our assumption of non-negative reporting delays is consistent with the literature [3, 14]. Fourth, as mentioned earlier, there are mainly three reasons for having missing symptom onset dates, i.e., they are missing due to human/system errors, pre-symptomatic cases or asymptomatic cases. By using negative binomial distribution for reporting delays, our model assumes the missing symptom onset dates are mostly due to human/system errors, which potentially biases the epidemic curve estimates as pre-symptomatic and asymptomatic cases could be substantial in COVID-19. However, the reproduction number estimates won’t suffer much from this if the proportion of pre-symptomatic and asymptomatic cases does not fluctuate much over time. Future work is needed for incorporating pre-symptomatic and asymptomatic cases into our modeling framework, potentially via labeling those two groups of cases and modeling negative reporting delays.

Overall, we provide a useful tool to estimate timely reproduction number estimates based on a Bayesian approach that integrates reporting delay imputation, back-calculation and nowcasting, all of which are interdependent and critical for reproduction number estimation. Our approach is robust to reasonable deviations from the model assumptions. Most importantly, it is more epidemiological meaningful than estimates based on the reported curve and thus a better option for surveillance of the COVID-19 pandemic.

## Supporting information

S1 Fig Impact of the maximum delay assumption for complete data when the reporting delay distribution was unchanged. For all graphs: the black solid curve corresponds to estimates based on the known epidemic curves and the black dashed curve corresponds to estimates based on the reported curves. The grey-shaded region superimposed on the curve depicts the 95% Bayesian credible interval and the grey-shade region on the right indicates the region of nowcasting. The colored curves represent different model choices. All values were averaged over 1000 simulated datasets.
(PDF)

S2 Fig Impact of the maximum delay assumption for complete data when the reporting delay distribution was sharply improved. For all graphs: the black solid curve corresponds to estimates based on the known epidemic curves and the black dashed curve corresponds to estimates based on the reported curves. The grey-shaded region superimposed on the curve depicts the 95% Bayesian credible interval and the grey-shade region on the right indicates the region of nowcasting. The colored curves represent different model choices. All values were averaged over 1000 simulated datasets.
(PDF)

S3 Fig Impact of the maximum delay assumption for complete data when the reporting delay distribution was gradually improved. For all graphs: the black solid curve corresponds to estimates based on the known epidemic curves and the black dashed curve corresponds to estimates based on the reported curves. The grey-shaded region superimposed on the curve depicts the 95% Bayesian credible interval and the grey-shade region on the right indicates the region of nowcasting. The colored curves represent different model choices. All values were averaged over 1000 simulated datasets.
(PDF)

S4 Fig Coverage rates of all estimated daily counts of symptom onset cases for complete data. For all graphs: The colored curves represent different model choices and the grey-shaded region indicates the nowcasting region. The coverage rates were calculated based on 1000 simulated datasets. A: The coverage rates given the reporting delay distribution was unchanged and *l* was correct. B: The coverage rates given the reporting delay distribution was unchanged and *l* was incorrect. C: The coverage rates given the reporting delay distribution was sharply improved and *l* was correct. D: The coverage rates given the reporting delay distribution was sharply improved and *l* was incorrect. E: The coverage rates given the reporting delay distribution was gradually improved and *l* was correct. F: The coverage rates given the reporting delay distribution was gradually improved and *l* was incorrect.
(PDF)

S5 Fig RMSE of all estimated daily counts of symptom onset cases for complete data. For all graphs: The colored curves represent different model choices and the grey-shaded region indicates the nowcasting region. The RMSE were calculated based on 1000 simulated datasets. A: The RMSE given the reporting delay distribution was unchanged and *l* was correct. B: The RMSE given the reporting delay distribution was unchanged and *l* was incorrect. C: The RMSE given the reporting delay distribution was sharply improved and *l* was correct. D: The RMSE given the reporting delay distribution was sharply improved and *l* was incorrect. E: The RMSE given the reporting delay distribution was gradually improved and *l* was correct. F: The RMSE given the reporting delay distribution was gradually improved and *l* was incorrect.
(PDF)

S6 Fig Coverage rates of all estimated daily counts of symptom onset cases for data with no early report. For all graphs: The colored curves represent different starting dates for line-list data and the grey-shaded region indicates the nowcasting region. The coverage rates were calculated based on 1000 simulated datasets. A: The coverage rates given the reporting delay distribution was unchanged and *l* was correct. B: The coverage rates given the reporting delay distribution was unchanged and *l* was incorrect. C: The coverage rates given the reporting delay distribution was sharply improved and *l* was correct. D: The coverage rates given the reporting delay distribution was sharply improved and *l* was incorrect. E: The coverage rates given the reporting delay distribution was gradually improved and *l* was correct. F: The coverage rates given the reporting delay distribution was gradually improved and *l* was incorrect.
(PDF)

S7 Fig RMSE of all estimated daily counts of symptom onset cases for data with no early report. For all graphs: The colored curves represent different starting dates for line-list data and the grey-shaded region indicates the nowcasting region. The RMSE were calculated based on 1000 simulated datasets. A: The RMSE given the reporting delay distribution was unchanged and *l* was correct. B: The RMSE given the reporting delay distribution was unchanged and *l* was incorrect. C: The RMSE given the reporting delay distribution was sharply improved and *l* was correct. D: The RMSE given the reporting delay distribution was sharply improved and *l* was incorrect. E: The RMSE given the reporting delay distribution was gradually improved and *l* was correct. F: The RMSE given the reporting delay distribution was gradually improved and *l* was incorrect.
(PDF)

S8 Fig Coverage rates of all estimated daily counts of symptom onset cases for an ongoing epidemic wave. For all graphs: The colored curves represent different ending dates for line-list data, and their nowcasting regions are displayed as the gray-shaded areas with boundary lines in their corresponding colors. The coverage rates were calculated based on 1000 simulated datasets. A: The coverage rates given the reporting delay distribution was unchanged and *l* was correct. B: The coverage rates given the reporting delay distribution was unchanged and *l* was incorrect. C: The coverage rates given the reporting delay distribution was sharply improved and *l* was correct. D: The coverage rates given the reporting delay distribution was sharply improved and *l* was incorrect. E: The coverage rates given the reporting delay distribution was gradually improved and *l* was correct. F: The coverage rates given the reporting delay distribution was gradually improved and *l* was incorrect.
(PDF)

S9 Fig RMSE of all estimated daily counts of symptom onset cases for an ongoing epidemic wave. For all graphs: The colored curves represent different ending dates for line-list data, and their nowcasting regions are displayed as the gray-shaded areas with boundary lines in their corresponding colors. The RMSE were calculated based on 1000 simulated datasets. A: The RMSE given the reporting delay distribution was unchanged and *l* was correct. B: The RMSE given the reporting delay distribution was unchanged and *l* was incorrect. C: The RMSE given the reporting delay distribution was sharply improved and *l* was correct. D: The RMSE given the reporting delay distribution was sharply improved and *l* was incorrect. E: The RMSE given the reporting delay distribution was gradually improved and *l* was correct. F: The RMSE given the reporting delay distribution was gradually improved and *l* was incorrect.
(PDF)

S1 Text More details on simulation design. (PDF)
