# Supplementary figures and images for: Bayesian back-calculation and nowcasting for line list data during the COVID-19 pandemic
Source: PLoS Comput Biol. 2021 Jul 12;17(7):e1009210. doi: 10.1371/journal.pcbi.1009210 (PMC8297945; doi:10.1371/journal.pcbi.1009210)

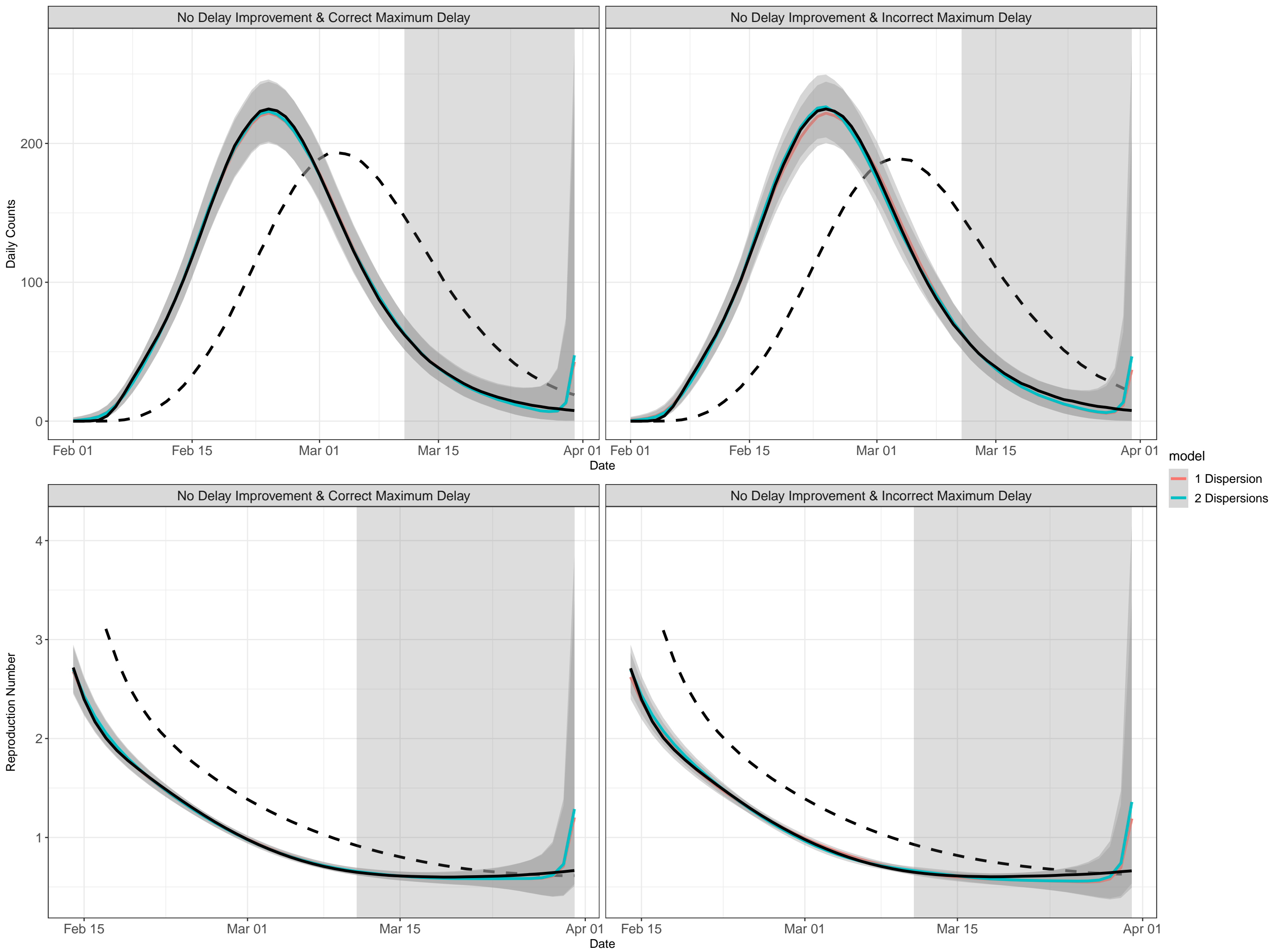

Supplement: S1 Fig — For all graphs: the black solid curve corresponds to estimates based on the known epidemic curves and the black dashed curve corresponds to estimates based on the reported curves. The grey-shaded region superimposed on the curve depicts the 95% Bayesian credible interval and the grey-shade region on the right indicates the region of nowcasting. The colored curves represent different model choices. All values were averaged over 1000 simulated datasets. (PDF) [file pcbi.1009210.s001.pdf]

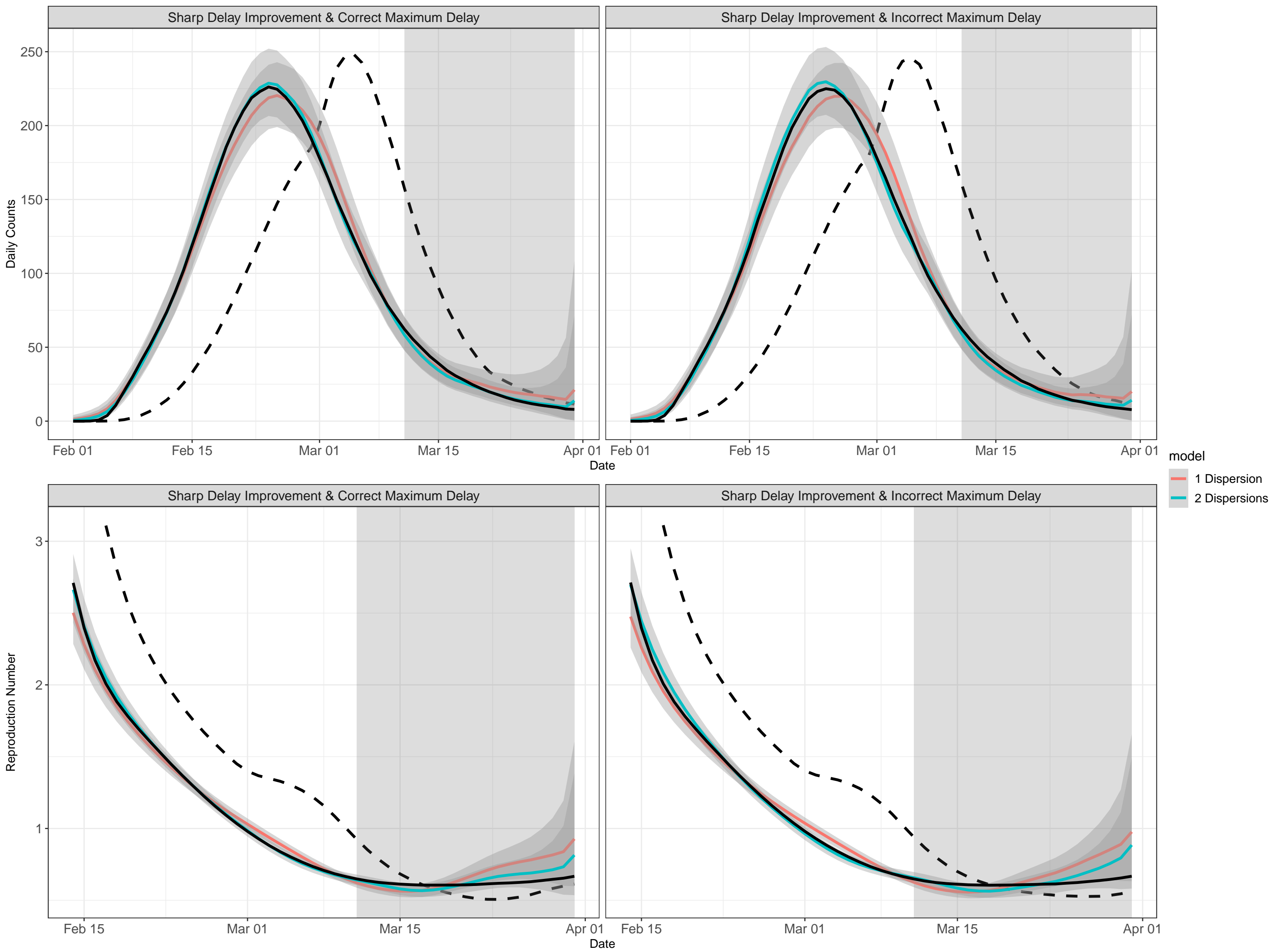

Supplement: S2 Fig — For all graphs: the black solid curve corresponds to estimates based on the known epidemic curves and the black dashed curve corresponds to estimates based on the reported curves. The grey-shaded region superimposed on the curve depicts the 95% Bayesian credible interval and the grey-shade region on the right indicates the region of nowcasting. The colored curves represent different model choices. All values were averaged over 1000 simulated datasets. (PDF) [file pcbi.1009210.s002.pdf]

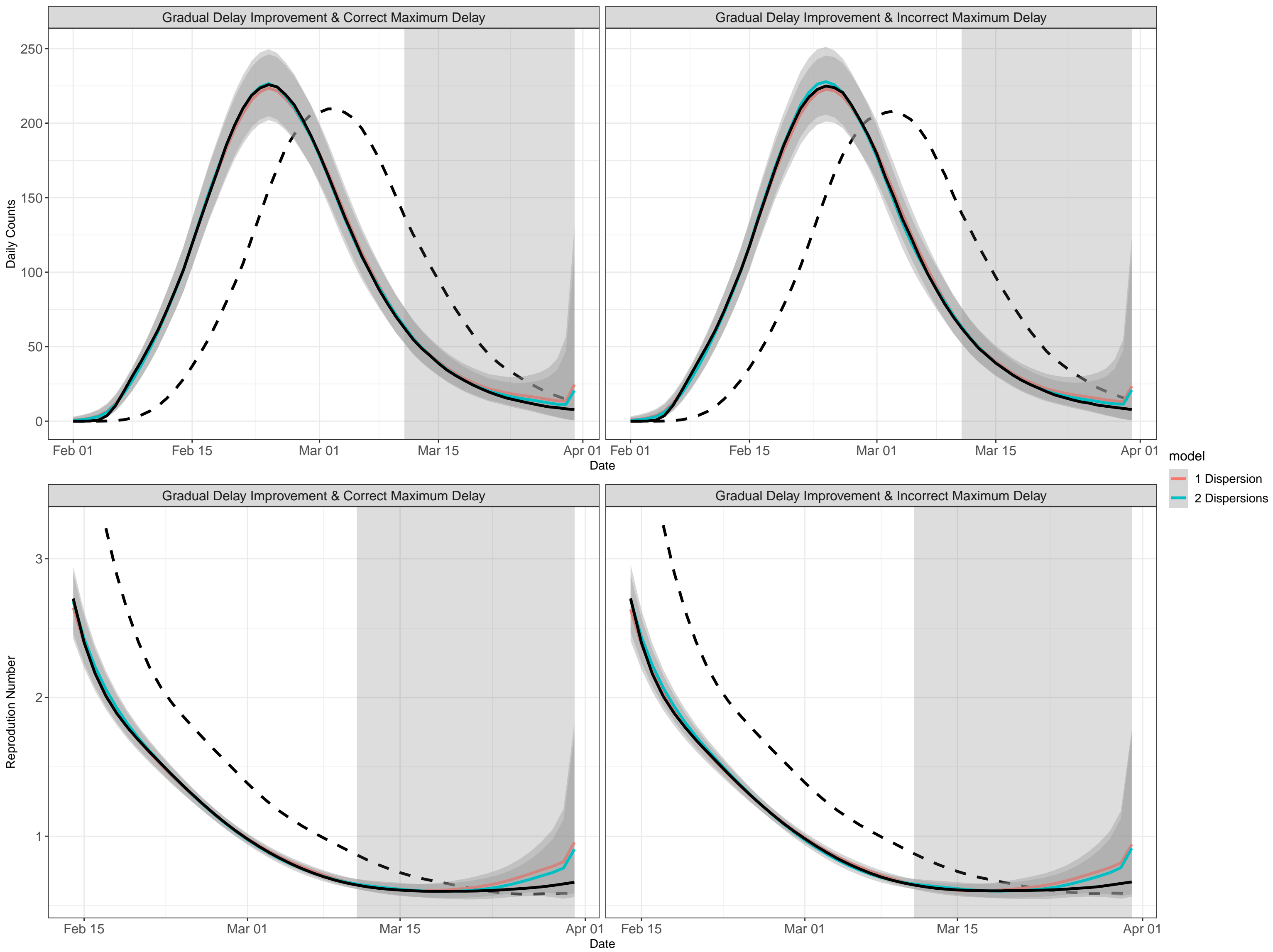

Supplement: S3 Fig — For all graphs: the black solid curve corresponds to estimates based on the known epidemic curves and the black dashed curve corresponds to estimates based on the reported curves. The grey-shaded region superimposed on the curve depicts the 95% Bayesian credible interval and the grey-shade region on the right indicates the region of nowcasting. The colored curves represent different model choices. All values were averaged over 1000 simulated datasets. (PDF) [file pcbi.1009210.s003.pdf]

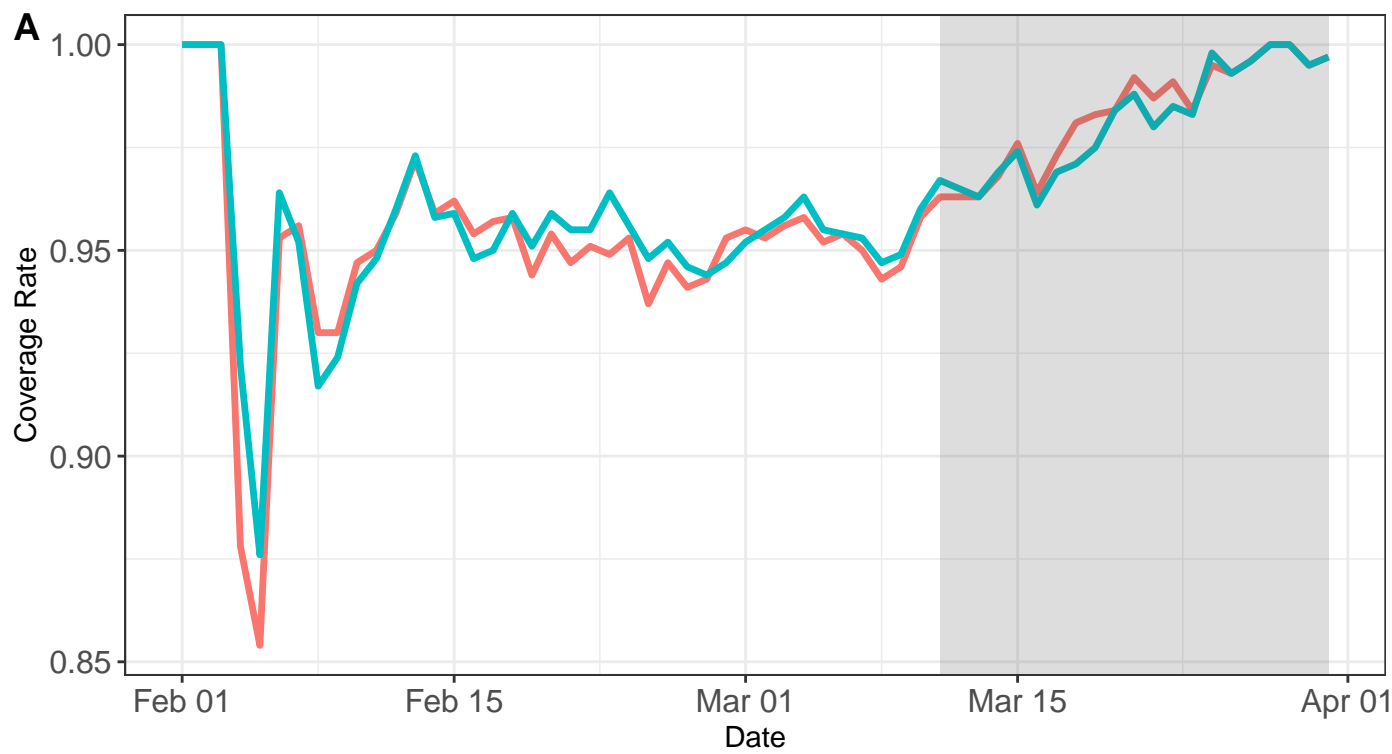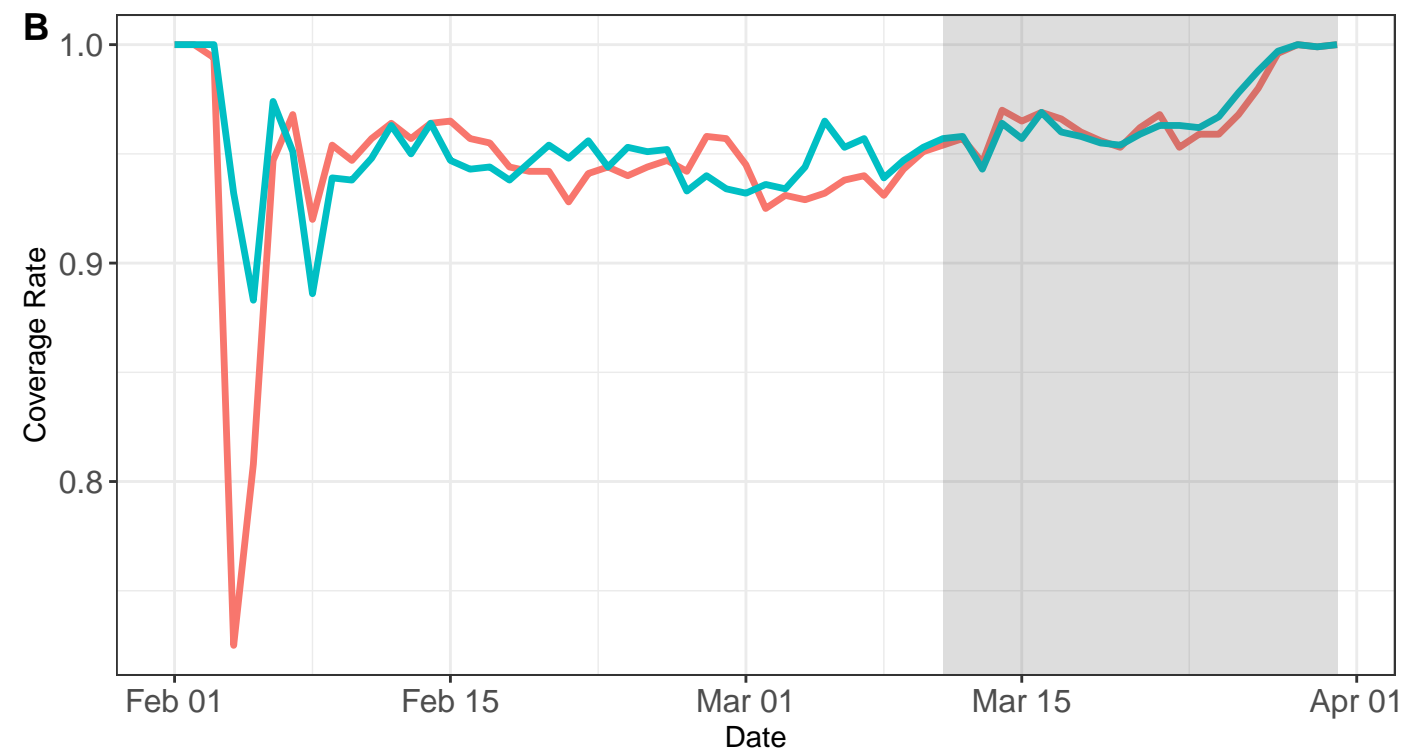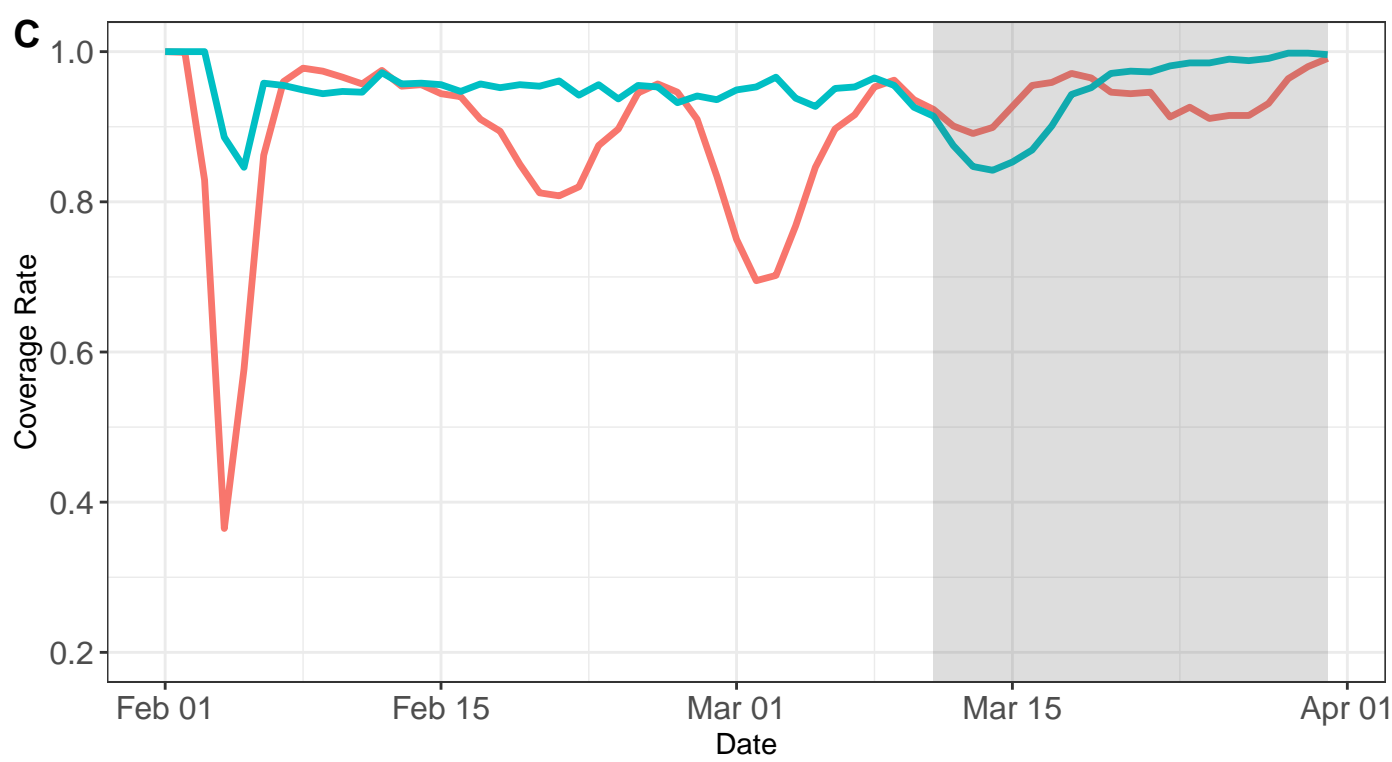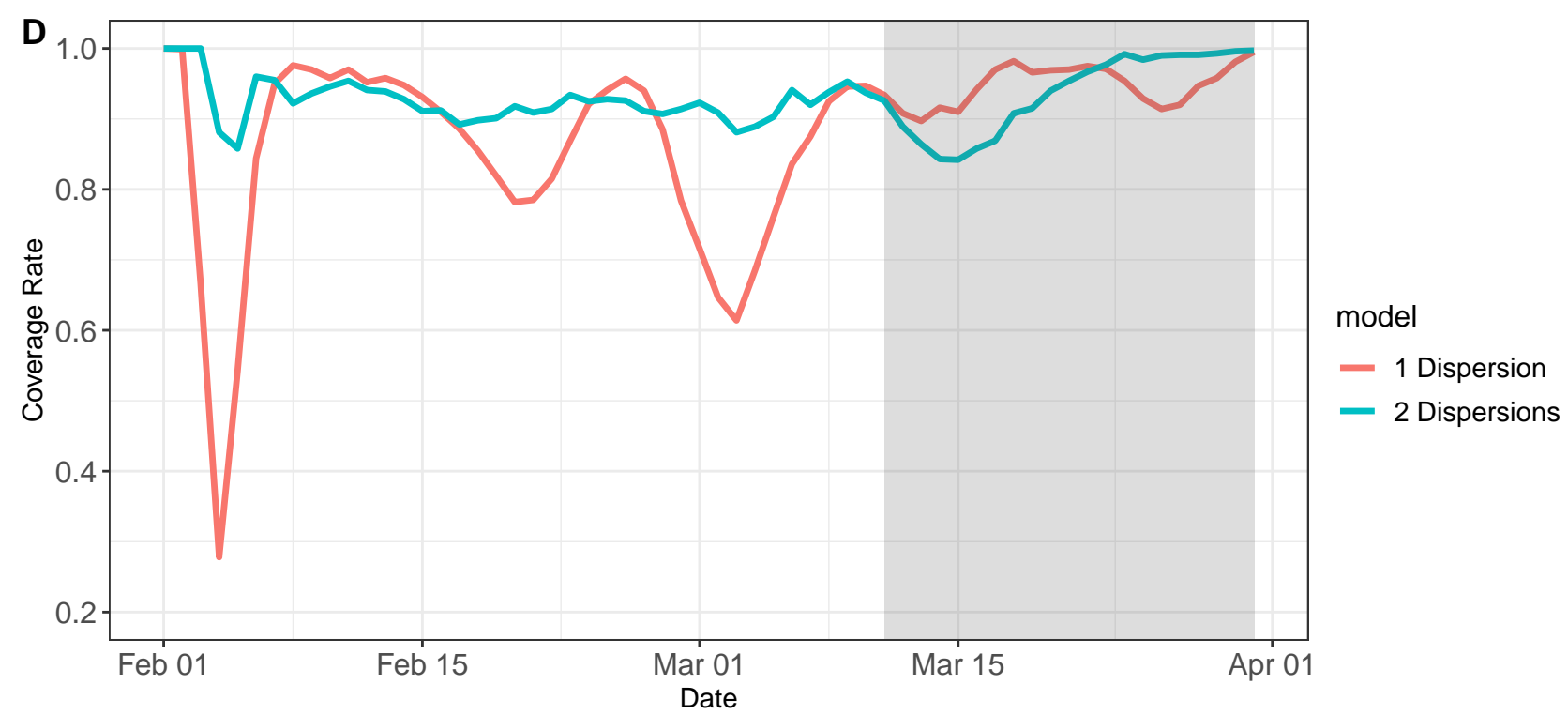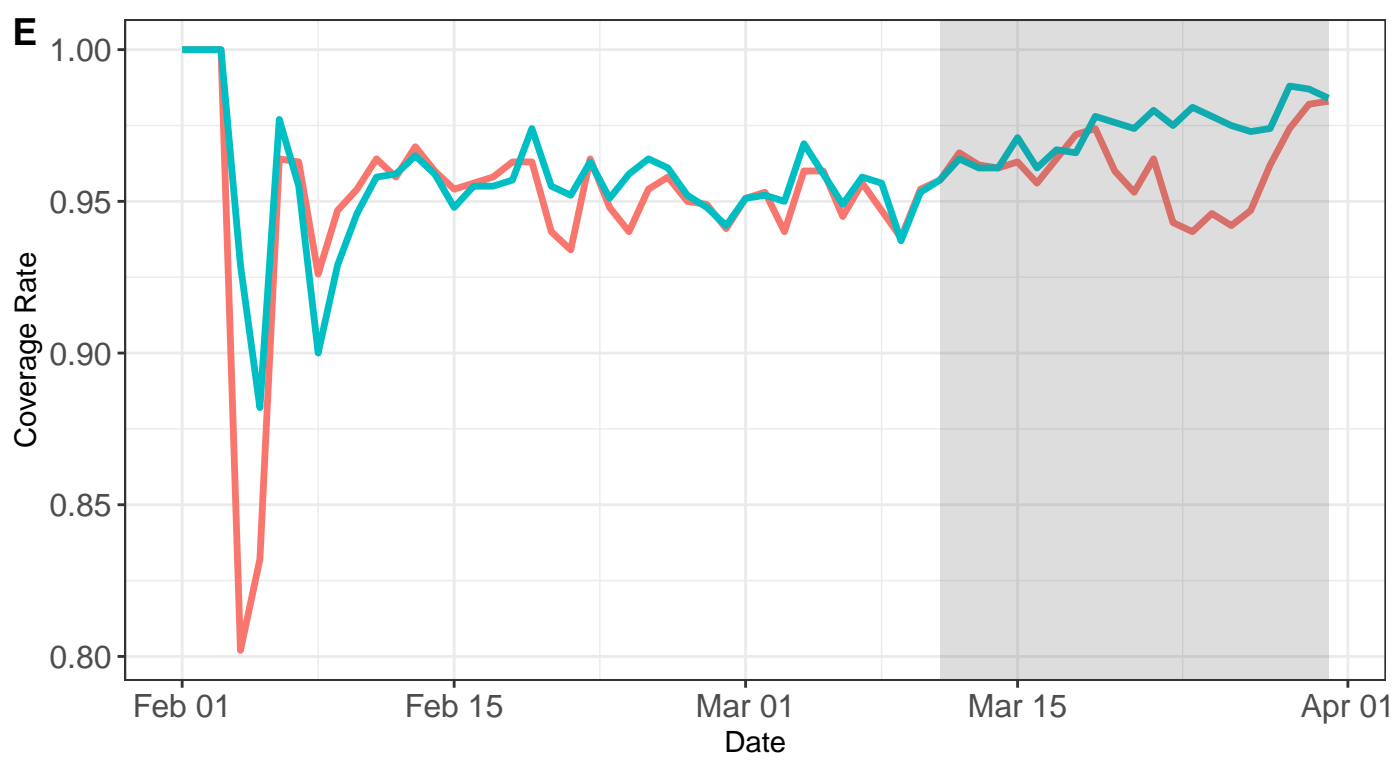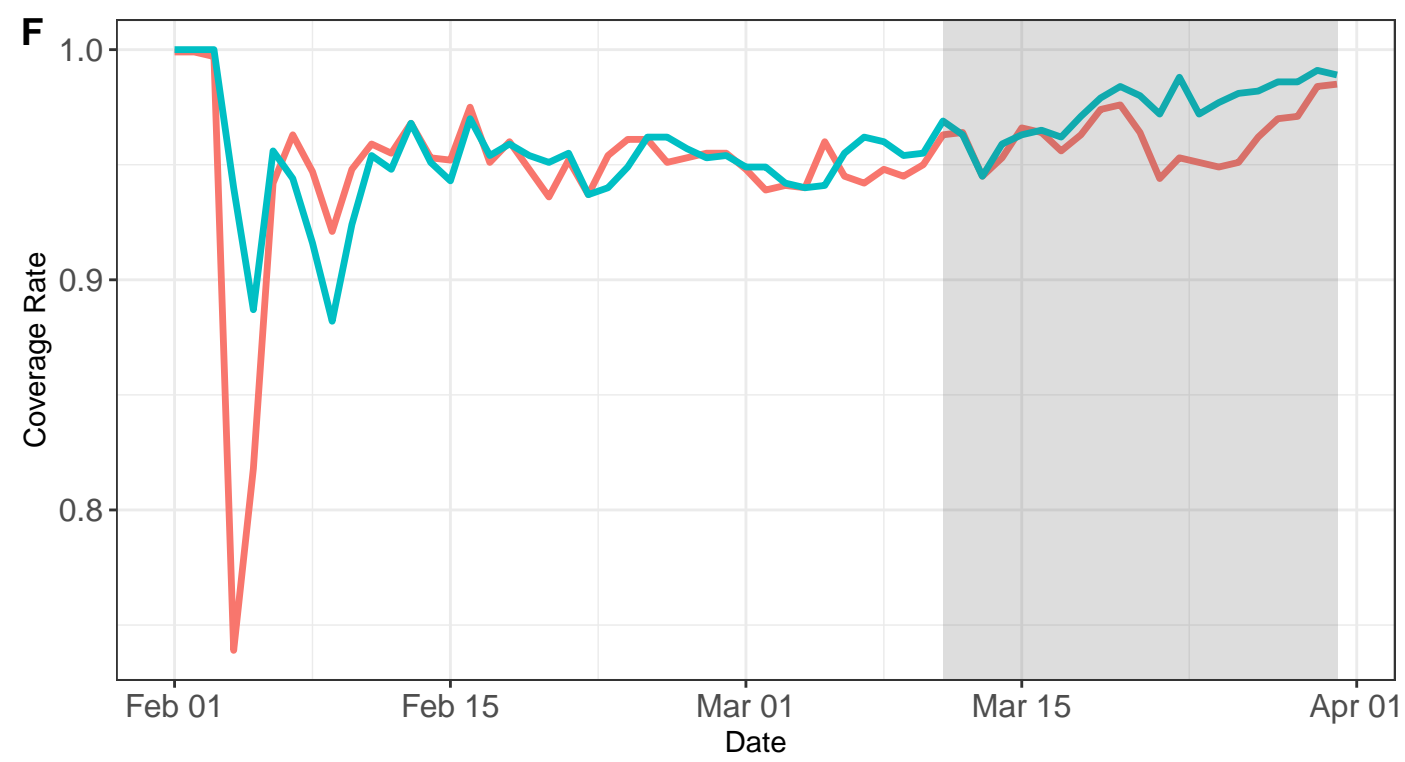

Supplement: S4 Fig — For all graphs: The colored curves represent different model choices and the grey-shaded region indicates the nowcasting region. The coverage rates were calculated based on 1000 simulated datasets. A: The coverage rates given the reporting delay distribution was unchanged and l was correct. B: The coverage rates given the reporting delay distribution was unchanged and l was incorrect. C: The coverage rates given the reporting delay distribution was sharply improved and l was correct. D: The coverage rates given the reporting delay distribution was sharply improved and l was incorrect. E: The coverage rates given the reporting delay distribution was gradually improved and l was correct. F: The coverage rates given the reporting delay distribution was gradually improved and l was incorrect. (PDF) [file pcbi.1009210.s004.pdf]

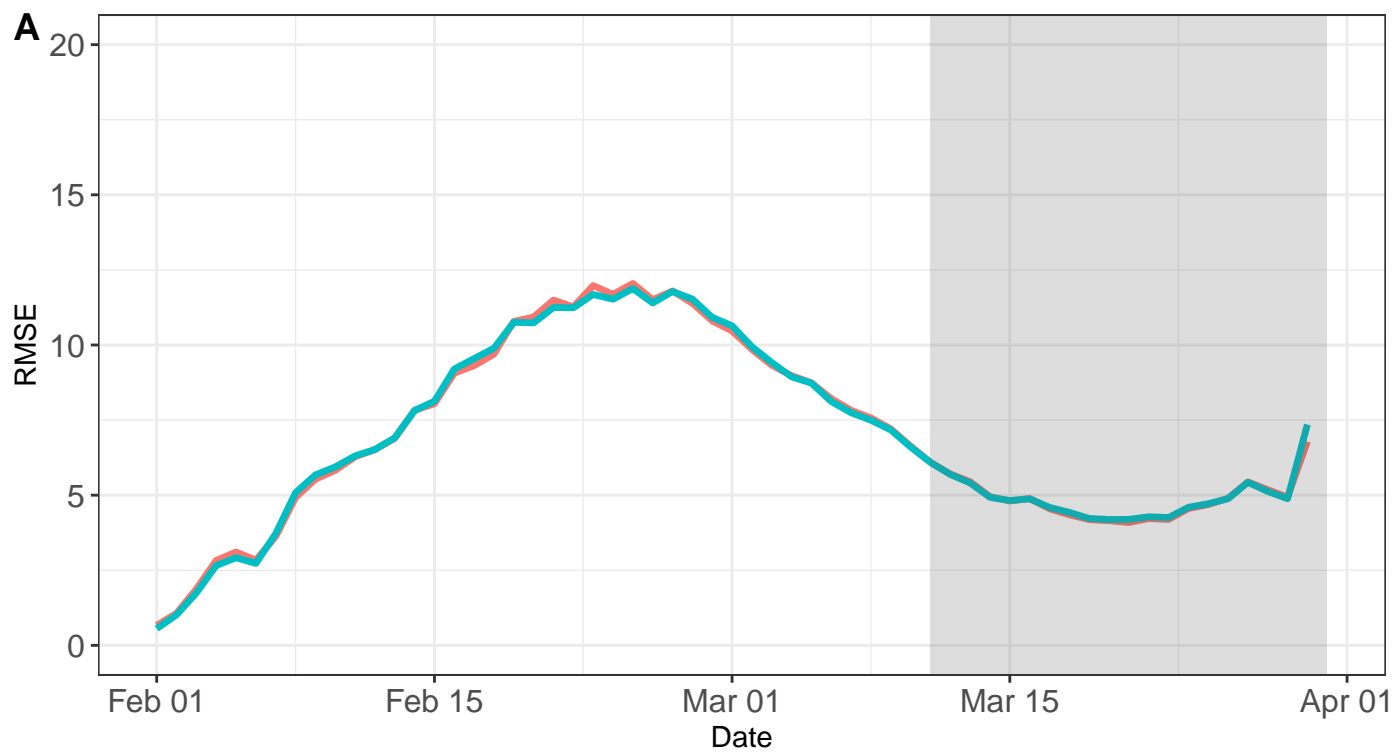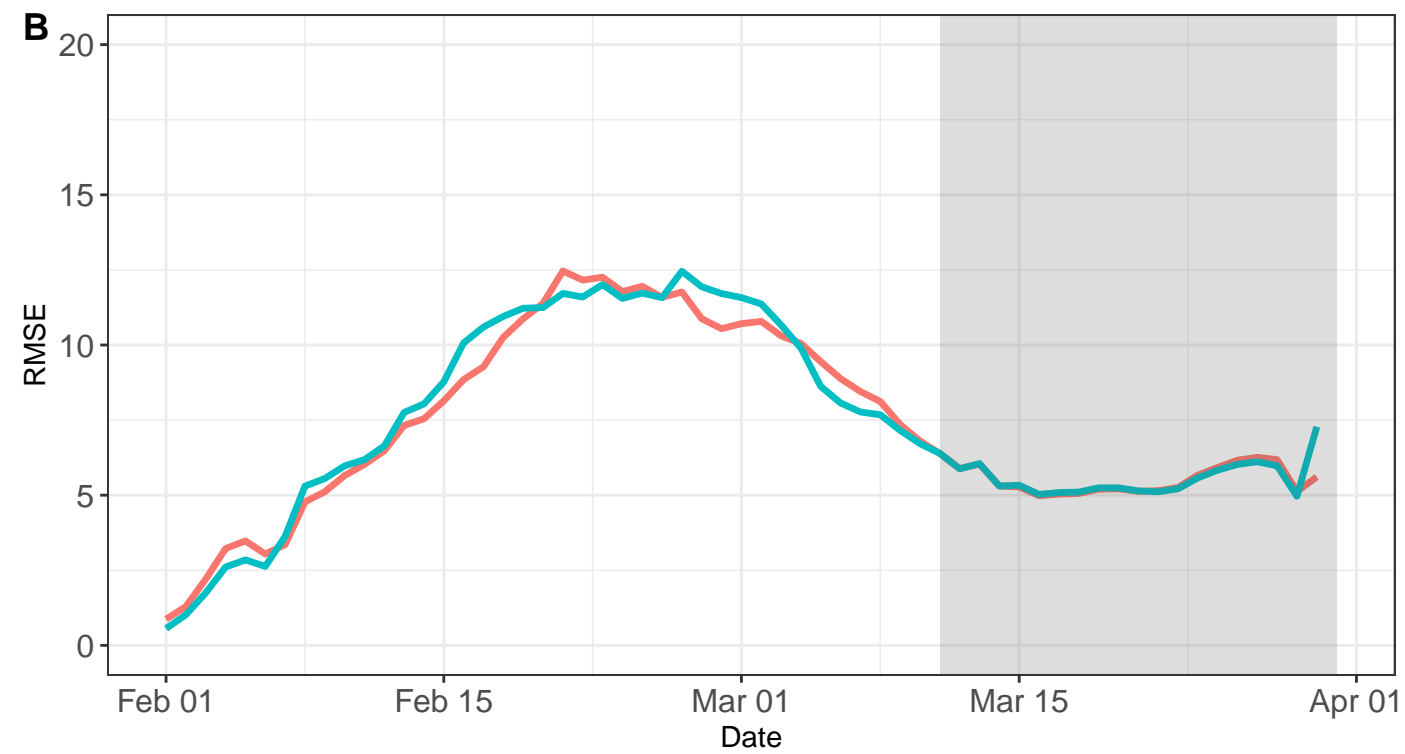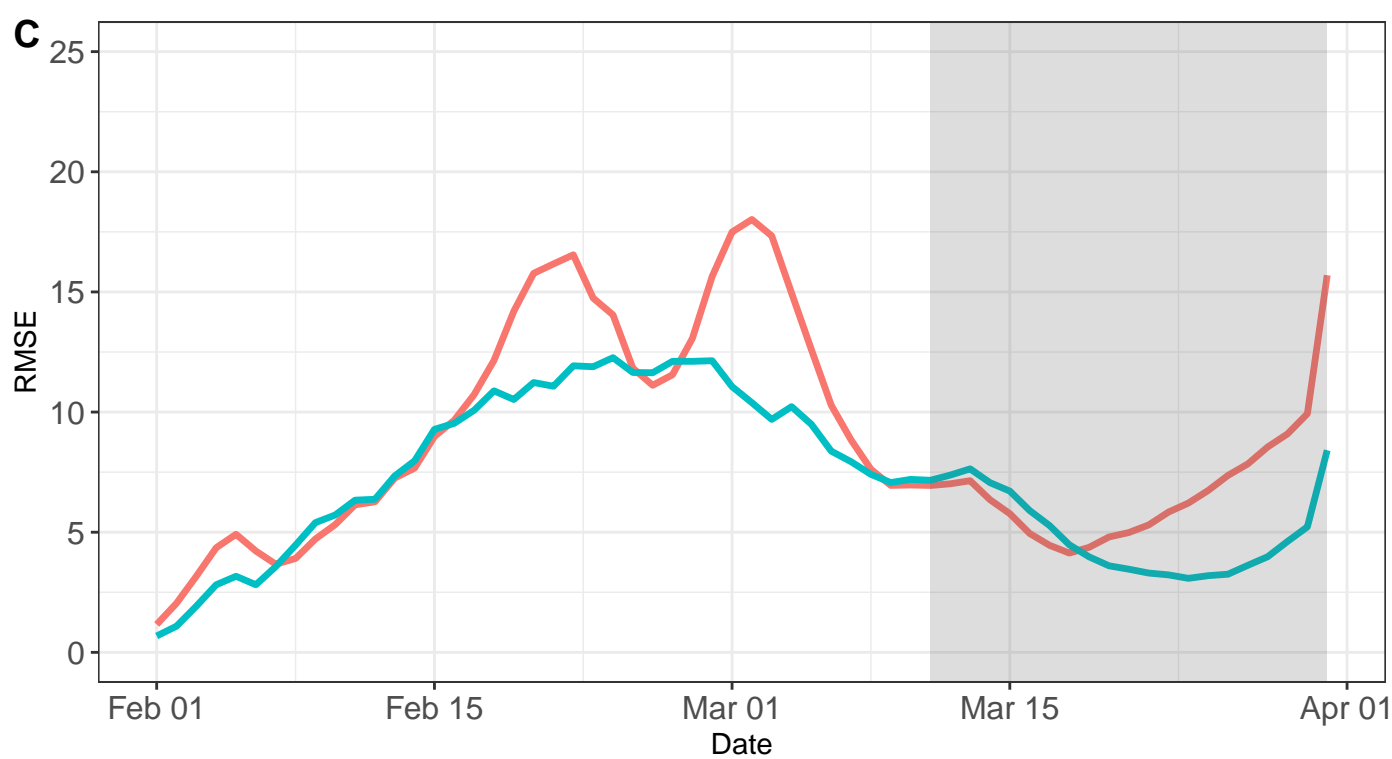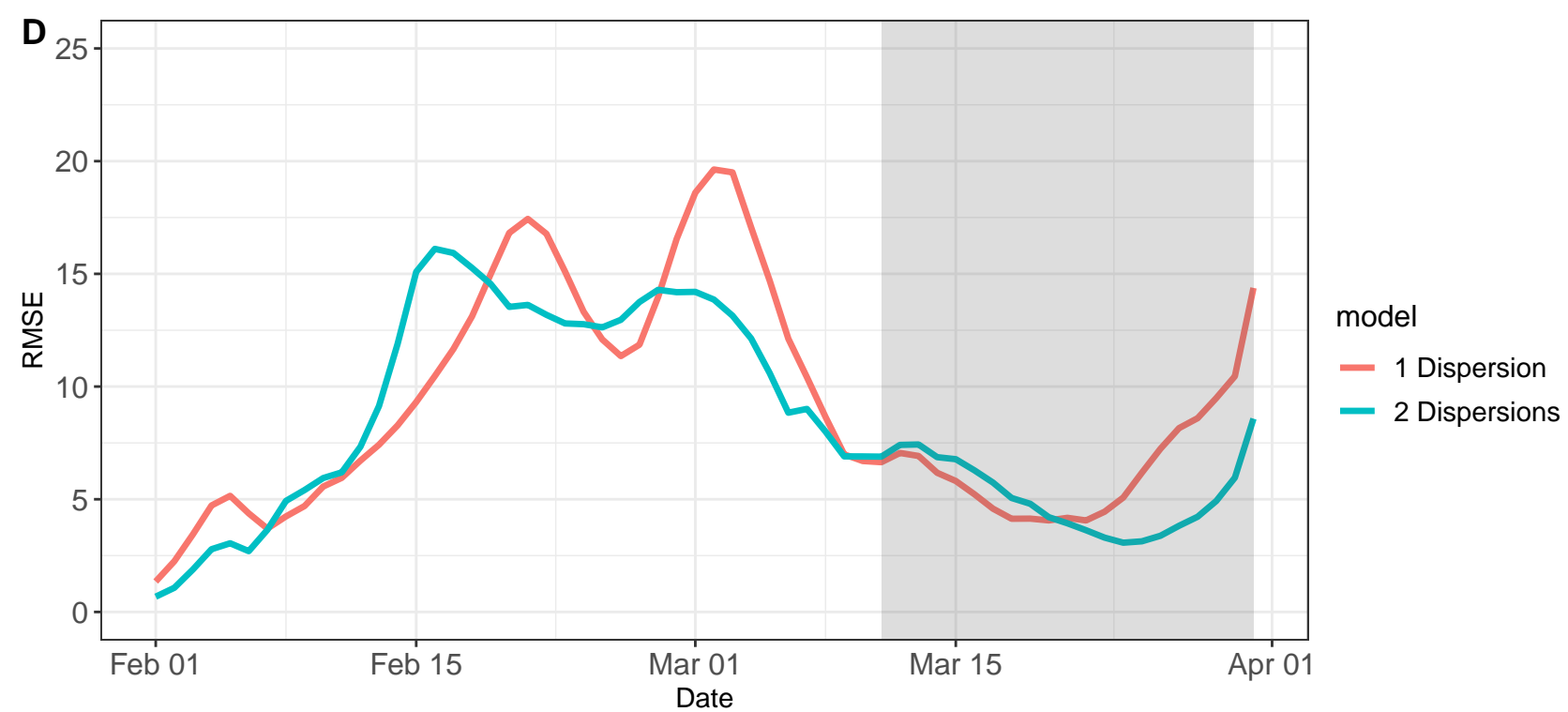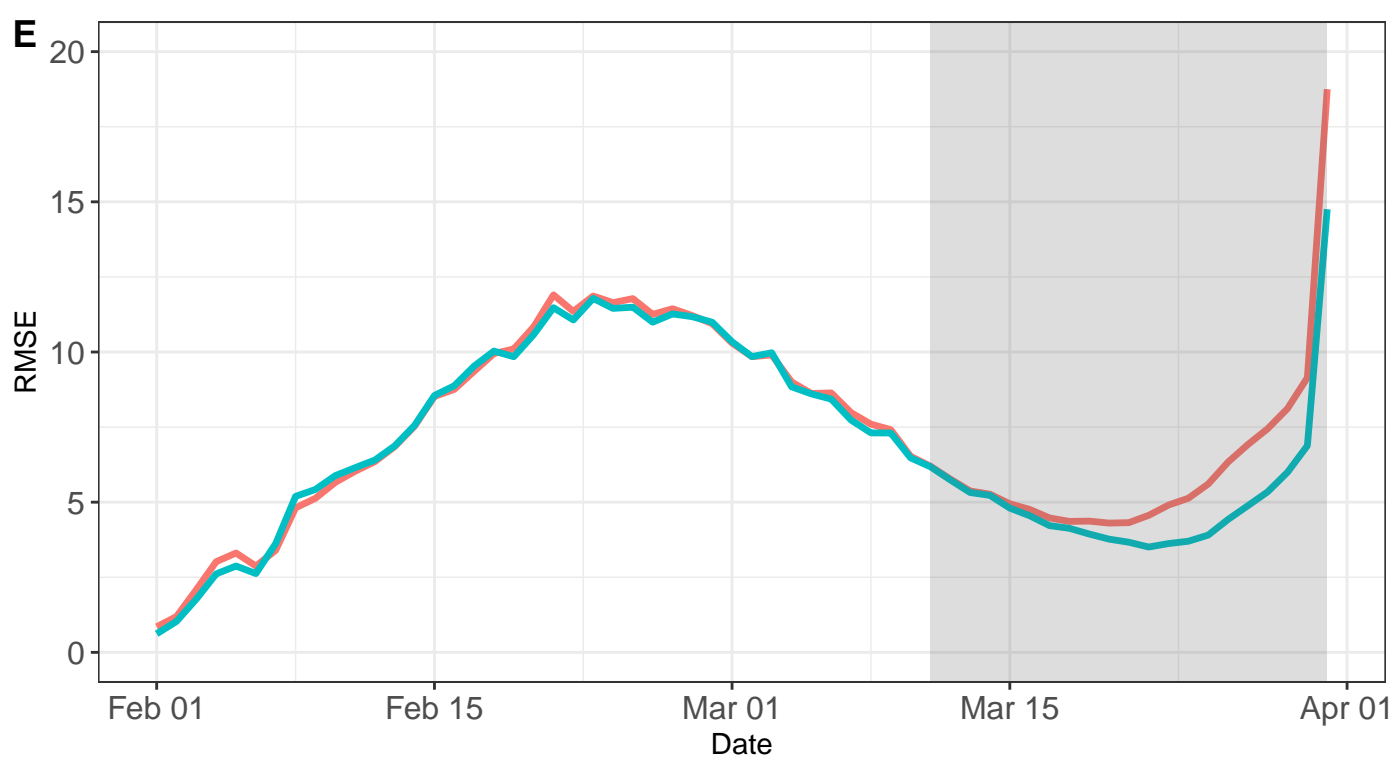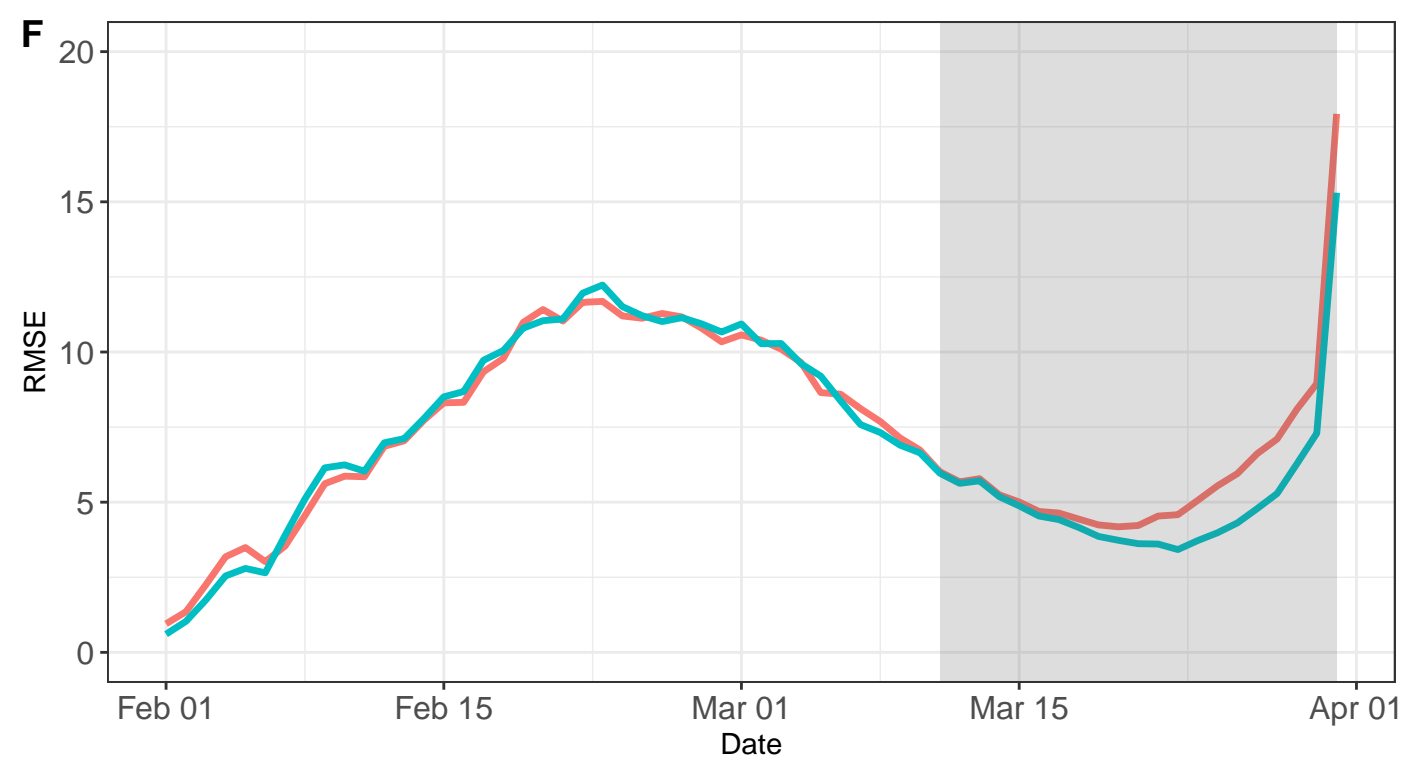

Supplement: S5 Fig — For all graphs: The colored curves represent different model choices and the grey-shaded region indicates the nowcasting region. The RMSE were calculated based on 1000 simulated datasets. A: The RMSE given the reporting delay distribution was unchanged and l was correct. B: The RMSE given the reporting delay distribution was unchanged and l was incorrect. C: The RMSE given the reporting delay distribution was sharply improved and l was correct. D: The RMSE given the reporting delay distribution was sharply improved and l was incorrect. E: The RMSE given the reporting delay distribution was gradually improved and l was correct. F: The RMSE given the reporting delay distribution was gradually improved and l was incorrect. (PDF) [file pcbi.1009210.s005.pdf]

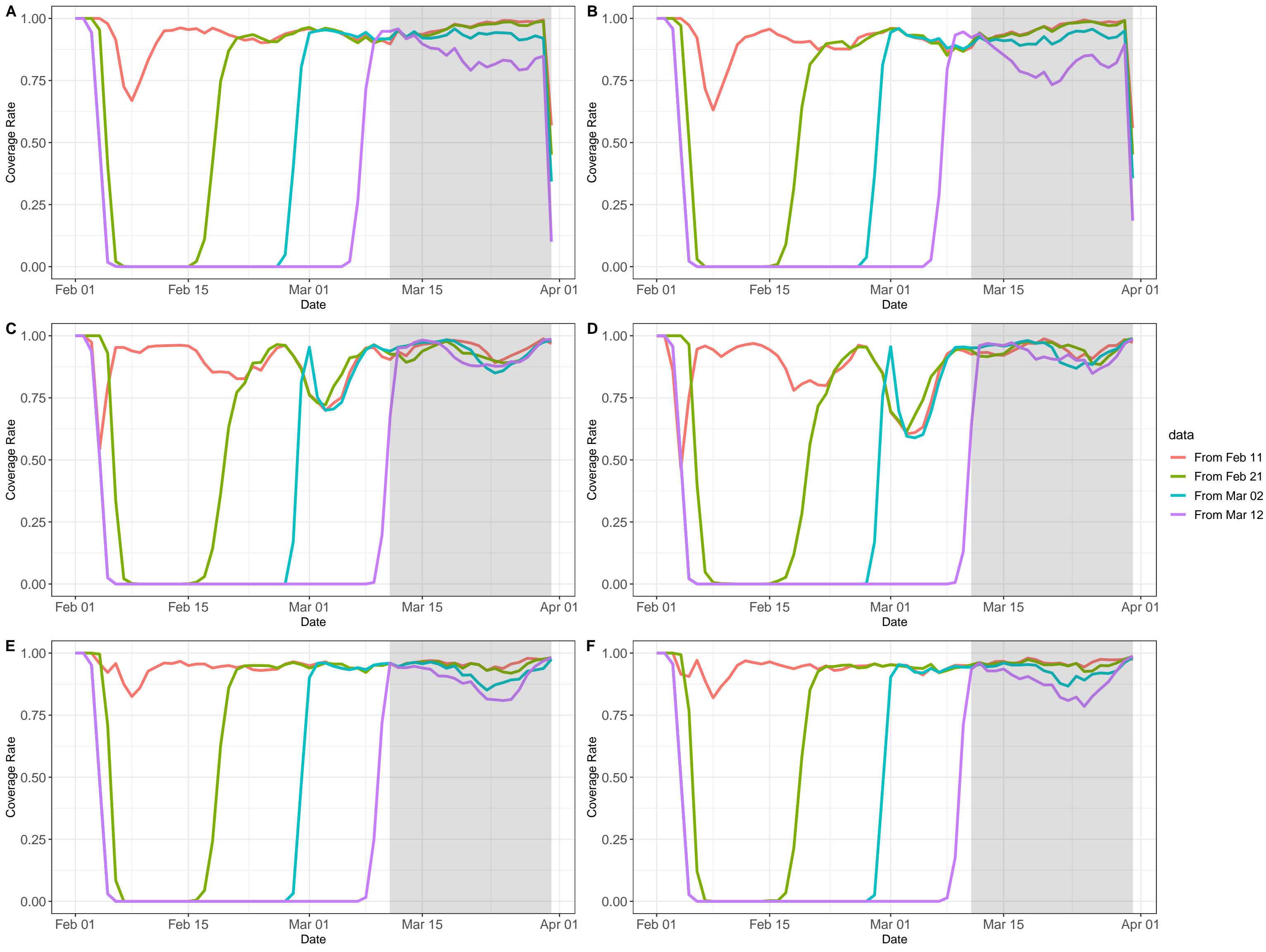

Supplement: S6 Fig — For all graphs: The colored curves represent different starting dates for line-list data and the grey-shaded region indicates the nowcasting region. The coverage rates were calculated based on 1000 simulated datasets. A: The coverage rates given the reporting delay distribution was unchanged and l was correct. B: The coverage rates given the reporting delay distribution was unchanged and l was incorrect. C: The coverage rates given the reporting delay distribution was sharply improved and l was correct. D: The coverage rates given the reporting delay distribution was sharply improved and l was incorrect. E: The coverage rates given the reporting delay distribution was gradually improved and l was correct. F: The coverage rates given the reporting delay distribution was gradually improved and l was incorrect. (PDF) [file pcbi.1009210.s006.pdf]

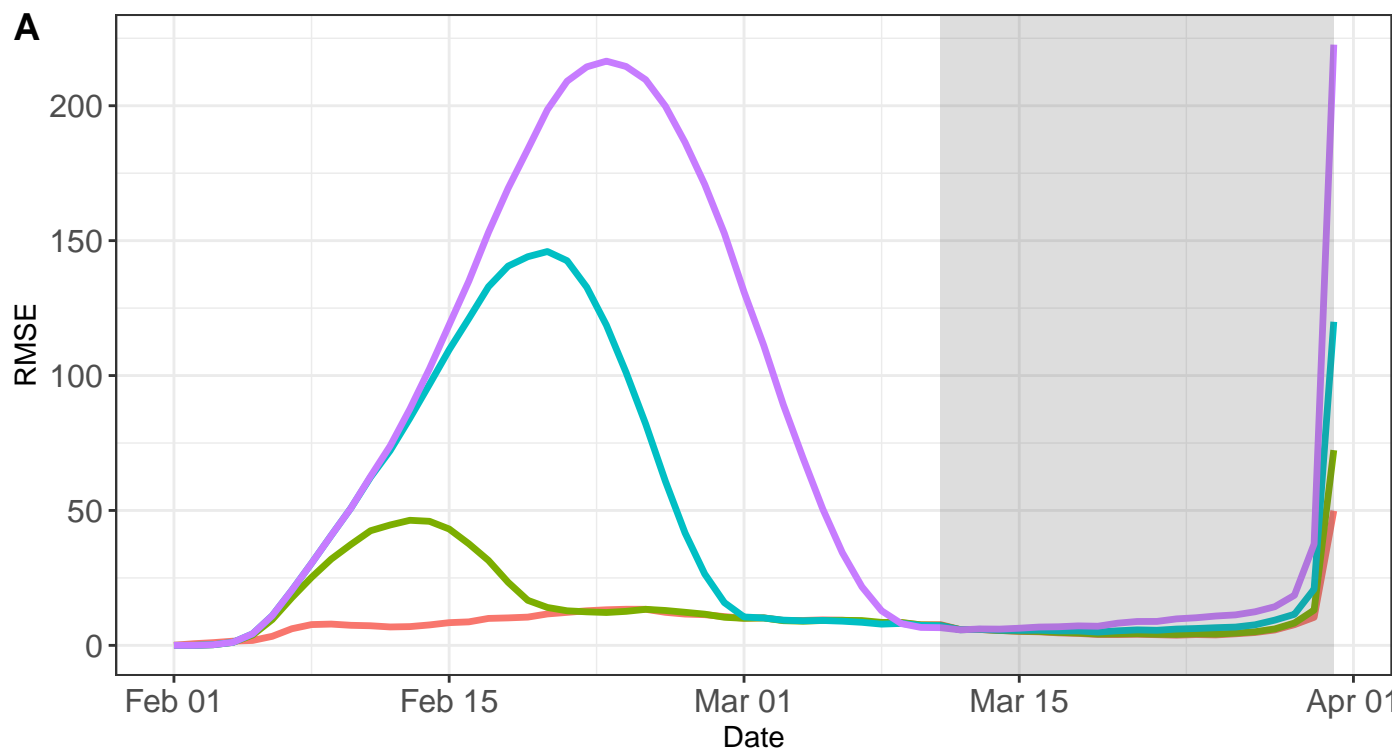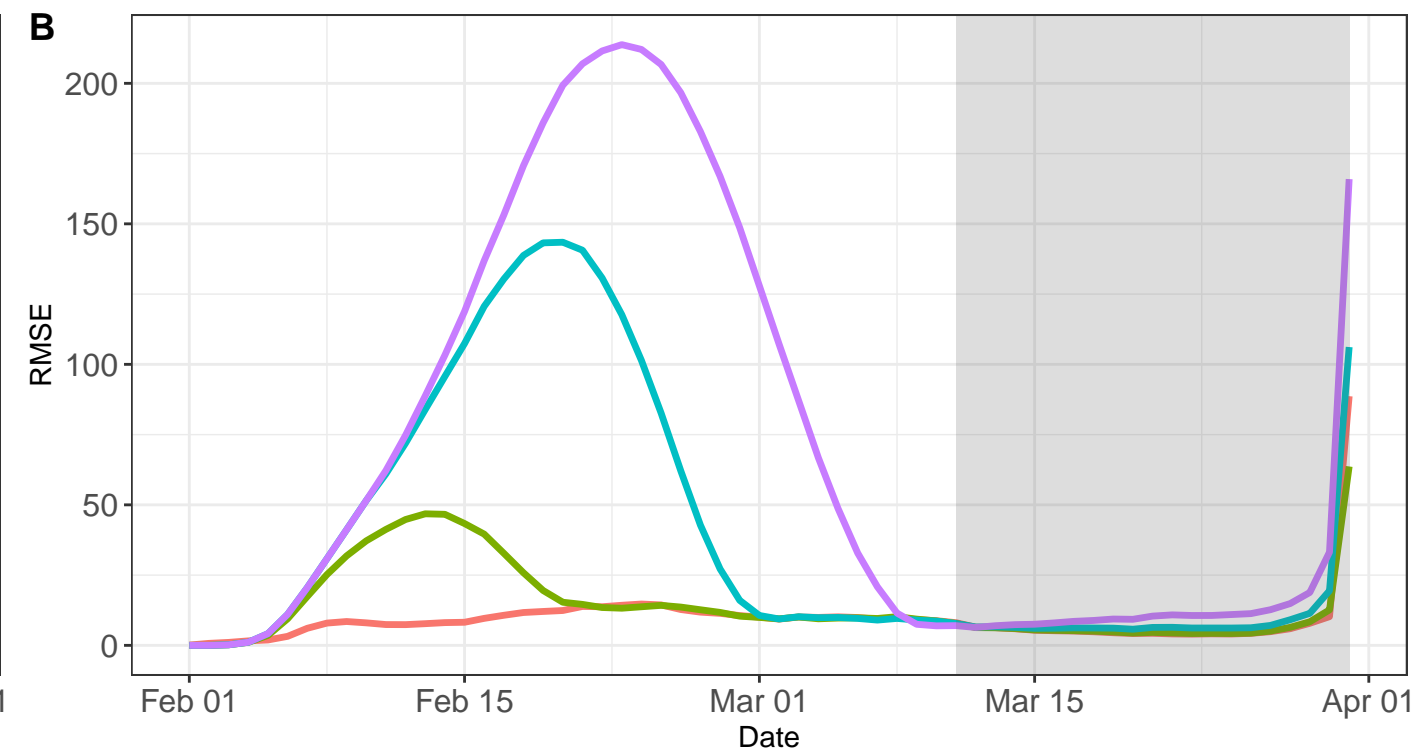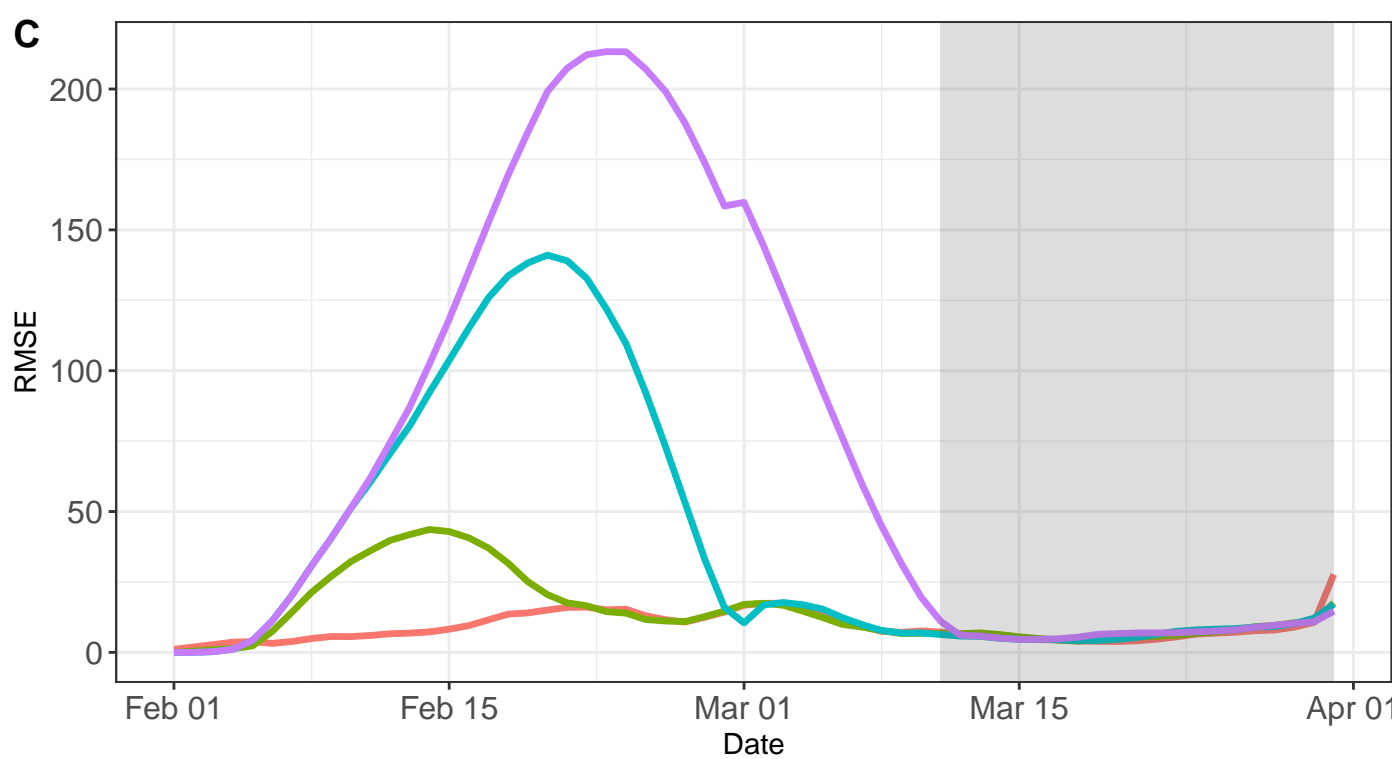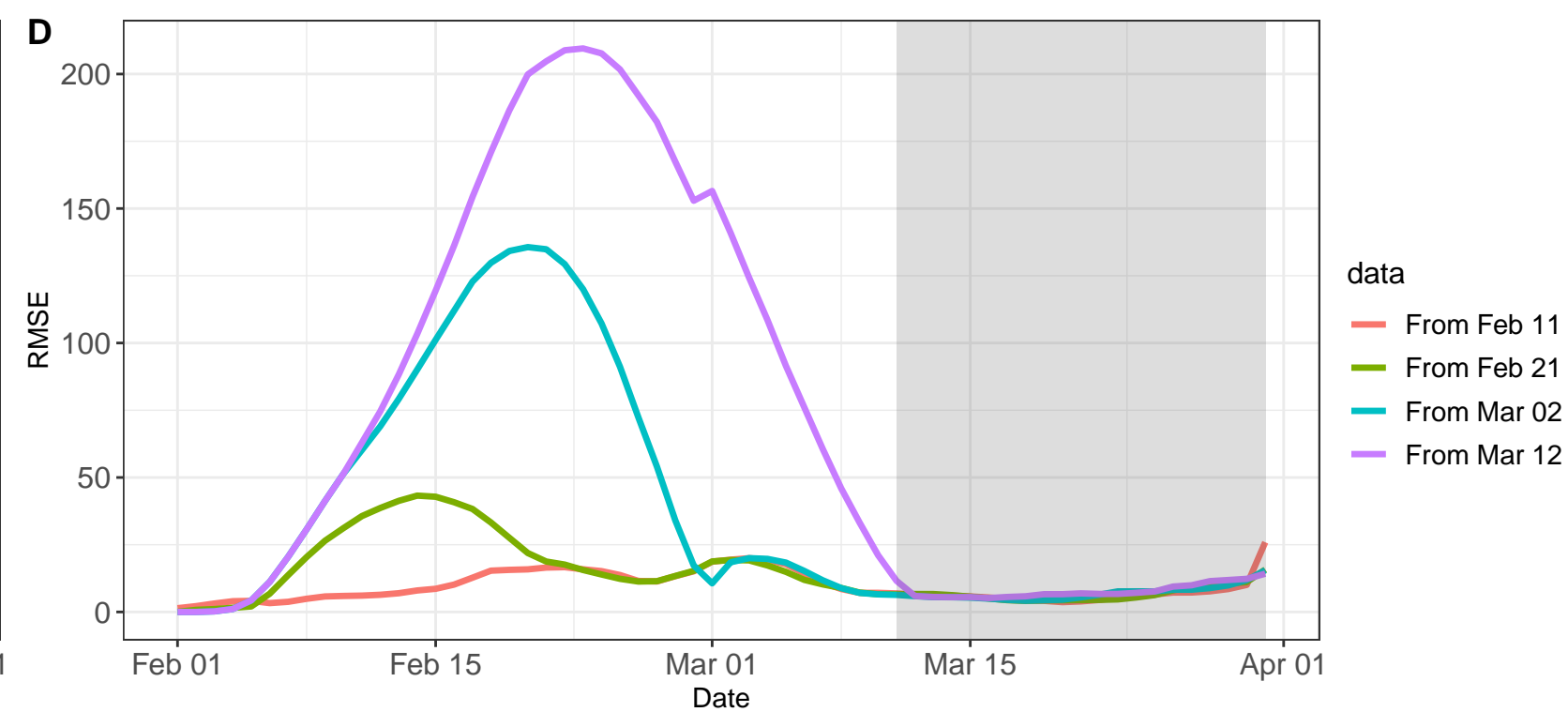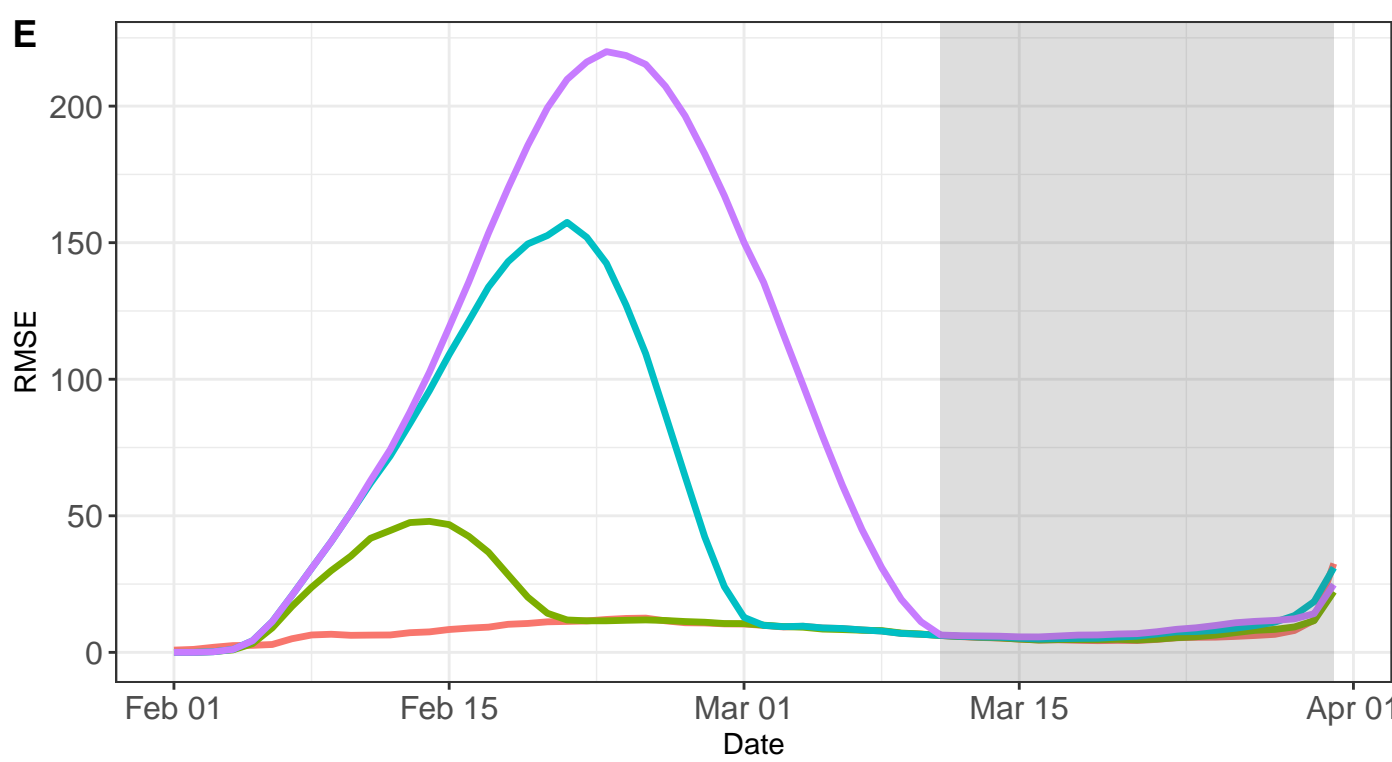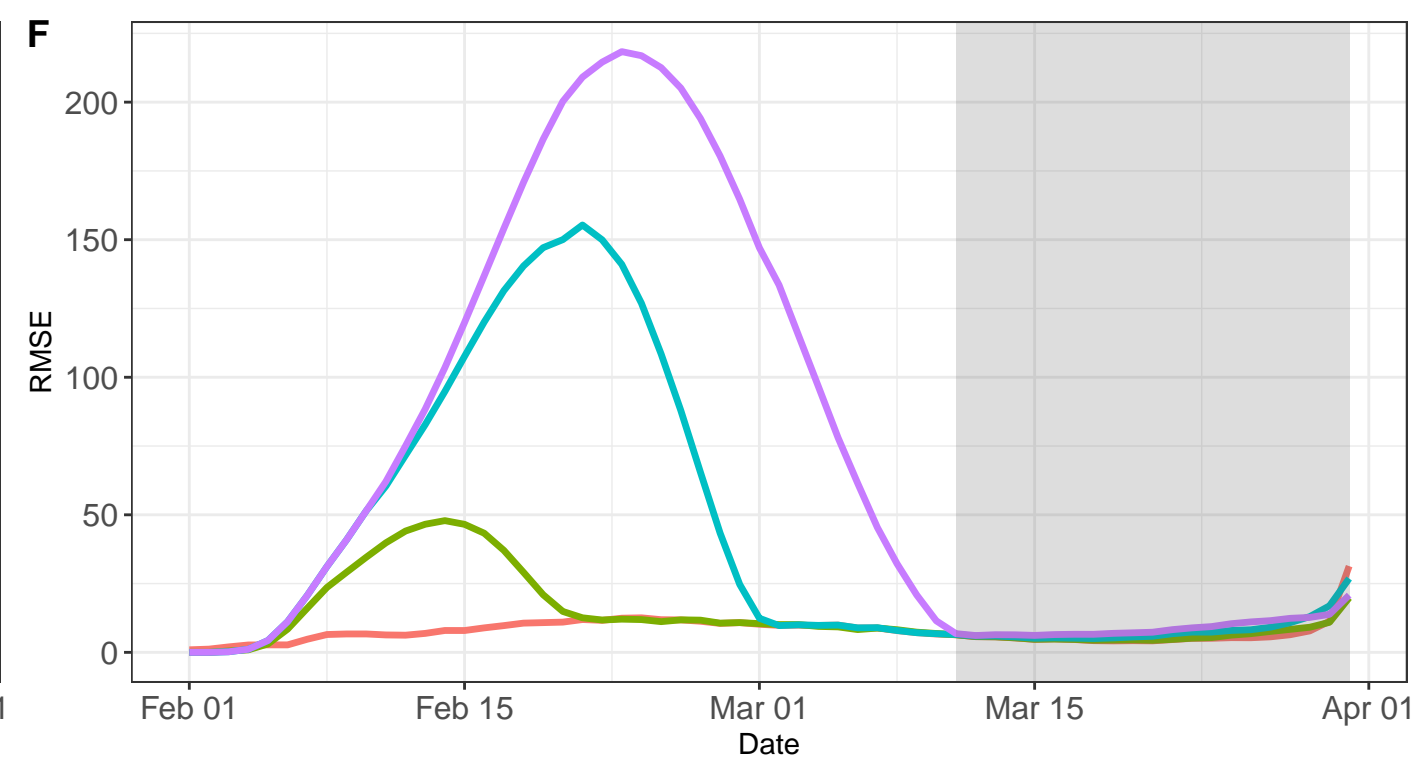

Supplement: S7 Fig — For all graphs: The colored curves represent different starting dates for line-list data and the grey-shaded region indicates the nowcasting region. The RMSE were calculated based on 1000 simulated datasets. A: The RMSE given the reporting delay distribution was unchanged and l was correct. B: The RMSE given the reporting delay distribution was unchanged and l was incorrect. C: The RMSE given the reporting delay distribution was sharply improved and l was correct. D: The RMSE given the reporting delay distribution was sharply improved and l was incorrect. E: The RMSE given the reporting delay distribution was gradually improved and l was correct. F: The RMSE given the reporting delay distribution was gradually improved and l was incorrect. (PDF) [file pcbi.1009210.s007.pdf]

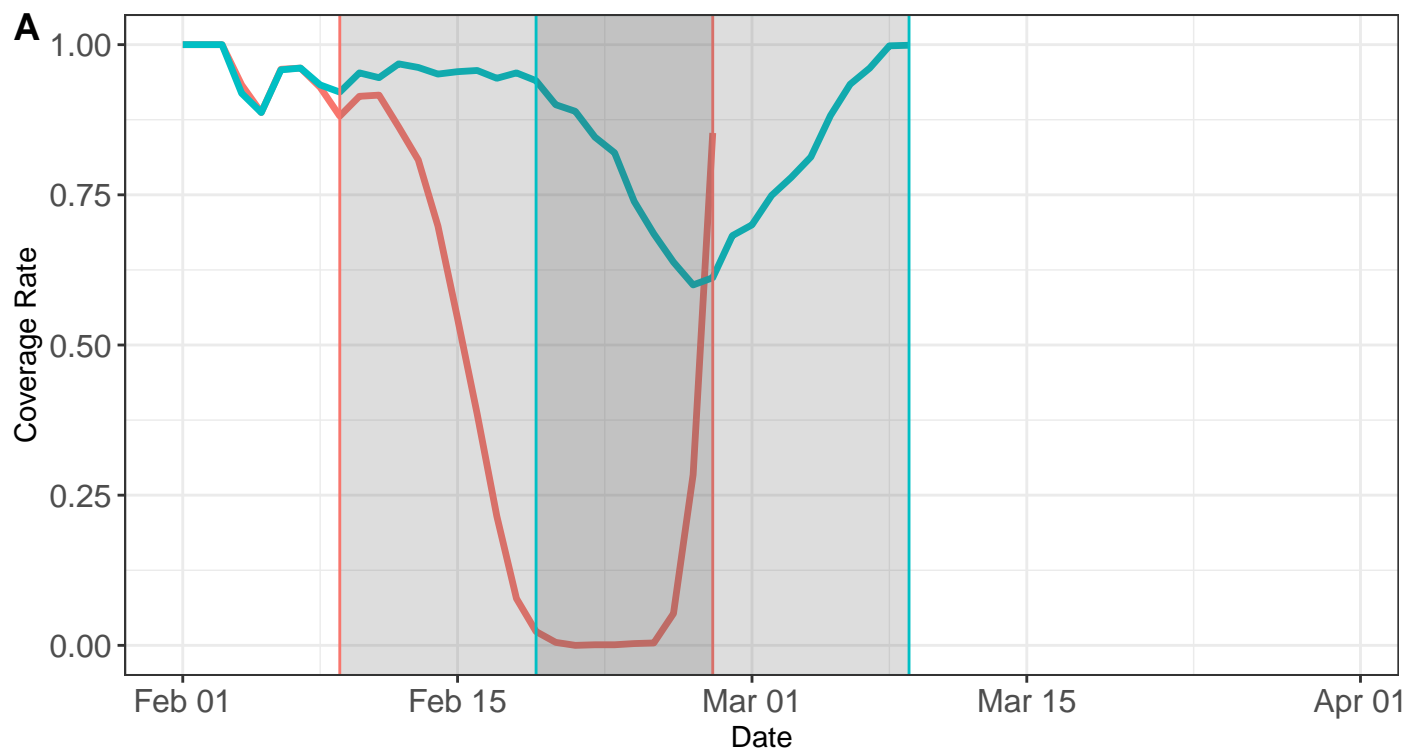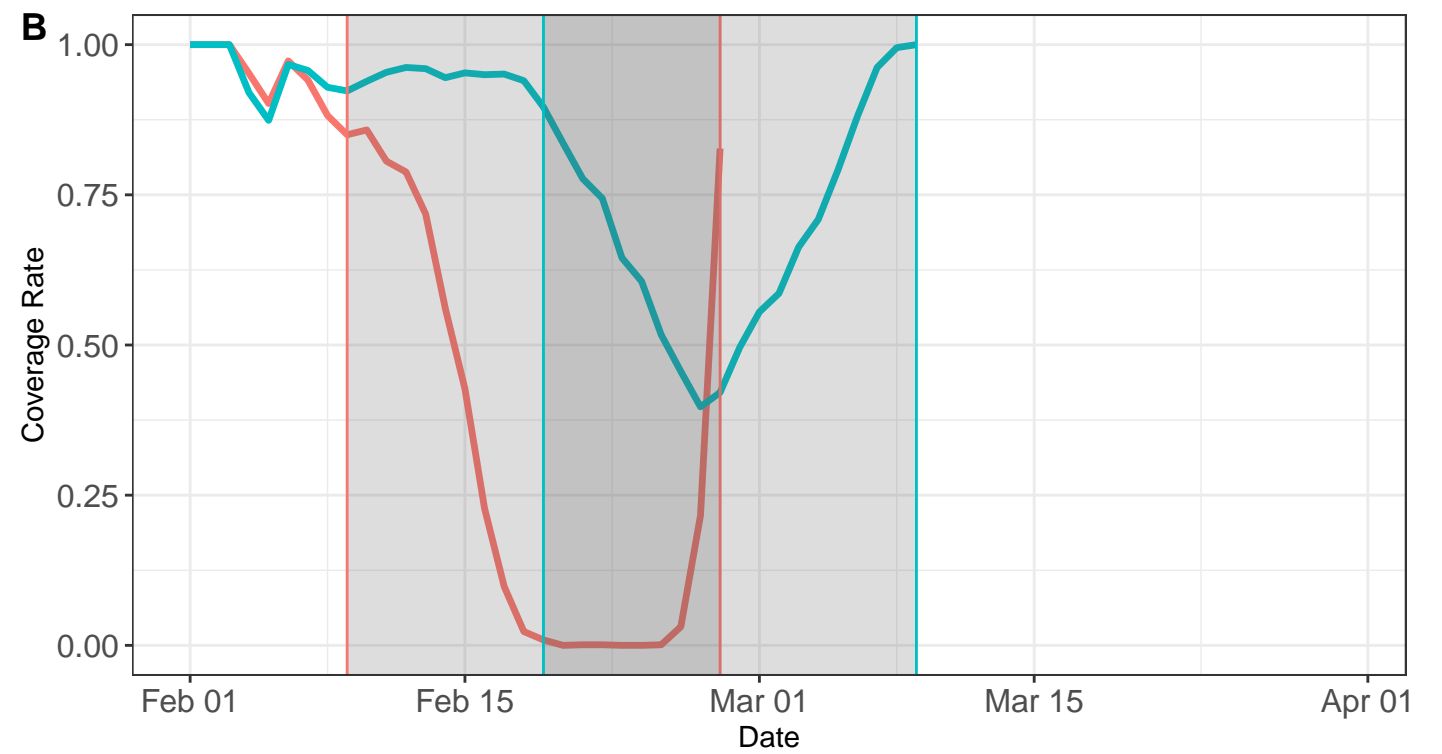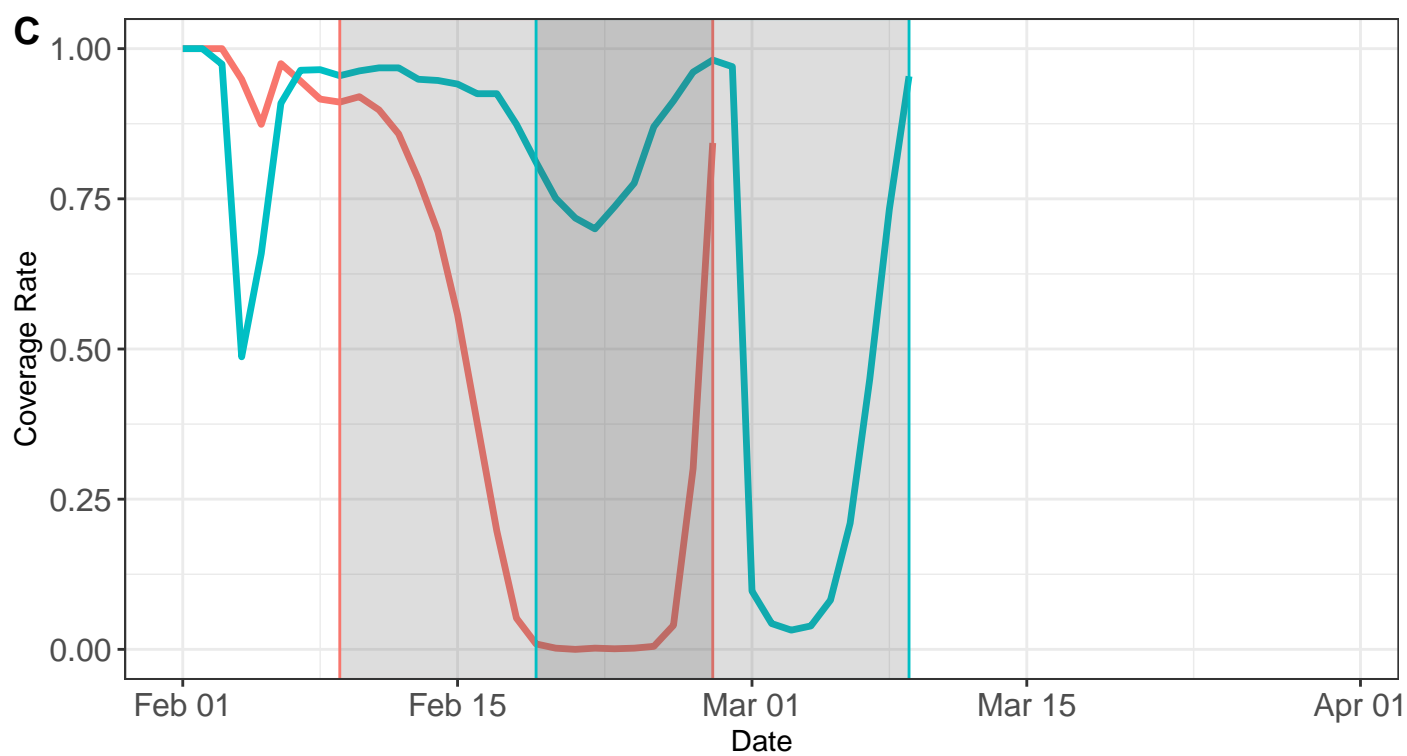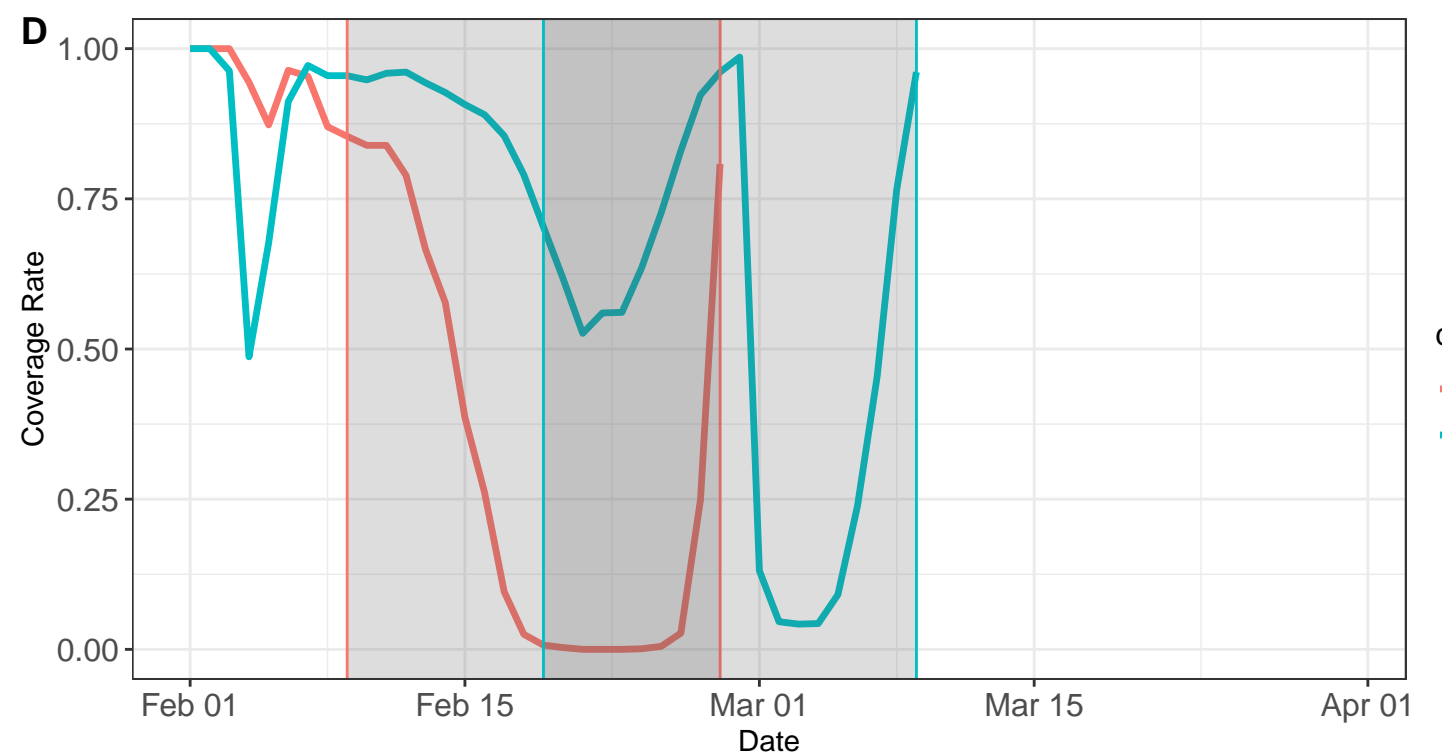

data

- Until Feb 28
- Until Mar 09

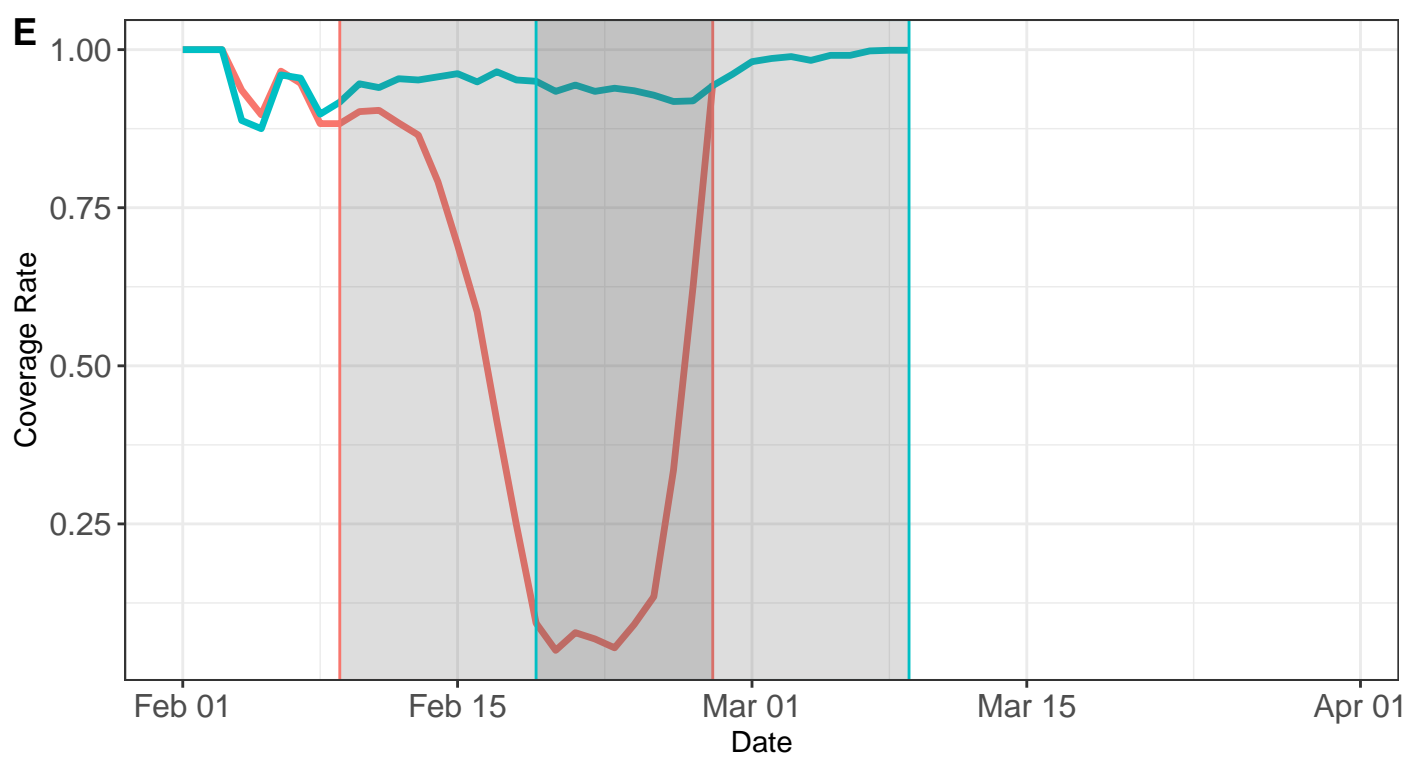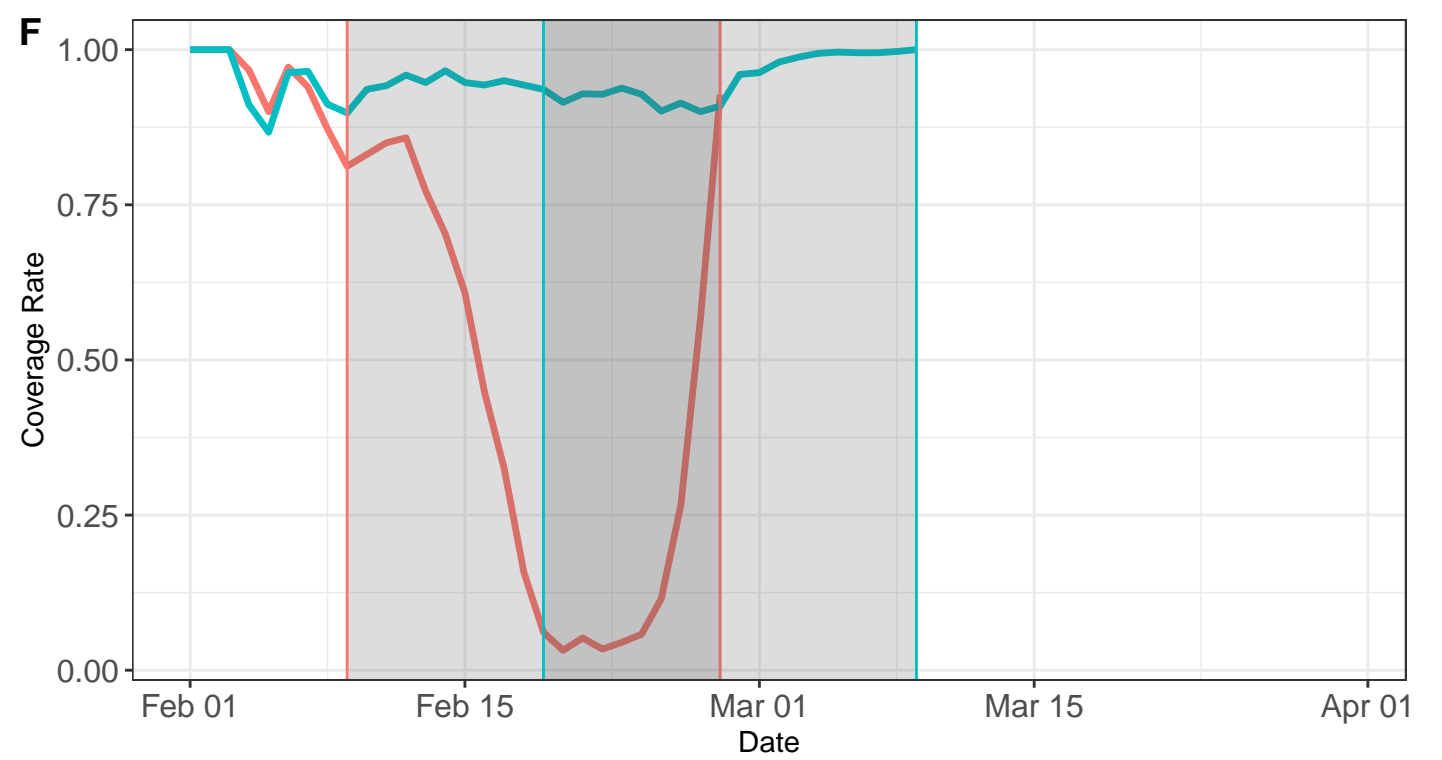

Supplement: S8 Fig — For all graphs: The colored curves represent different ending dates for line-list data, and their nowcasting regions are displayed as the gray-shaded areas with boundary lines in their corresponding colors. The coverage rates were calculated based on 1000 simulated datasets. A: The coverage rates given the reporting delay distribution was unchanged and l was correct. B: The coverage rates given the reporting delay distribution was unchanged and l was incorrect. C: The coverage rates given the reporting delay distribution was sharply improved and l was correct. D: The coverage rates given the reporting delay distribution was sharply improved and l was incorrect. E: The coverage rates given the reporting delay distribution was gradually improved and l was correct. F: The coverage rates given the reporting delay distribution was gradually improved and l was incorrect. (PDF) [file pcbi.1009210.s008.pdf]

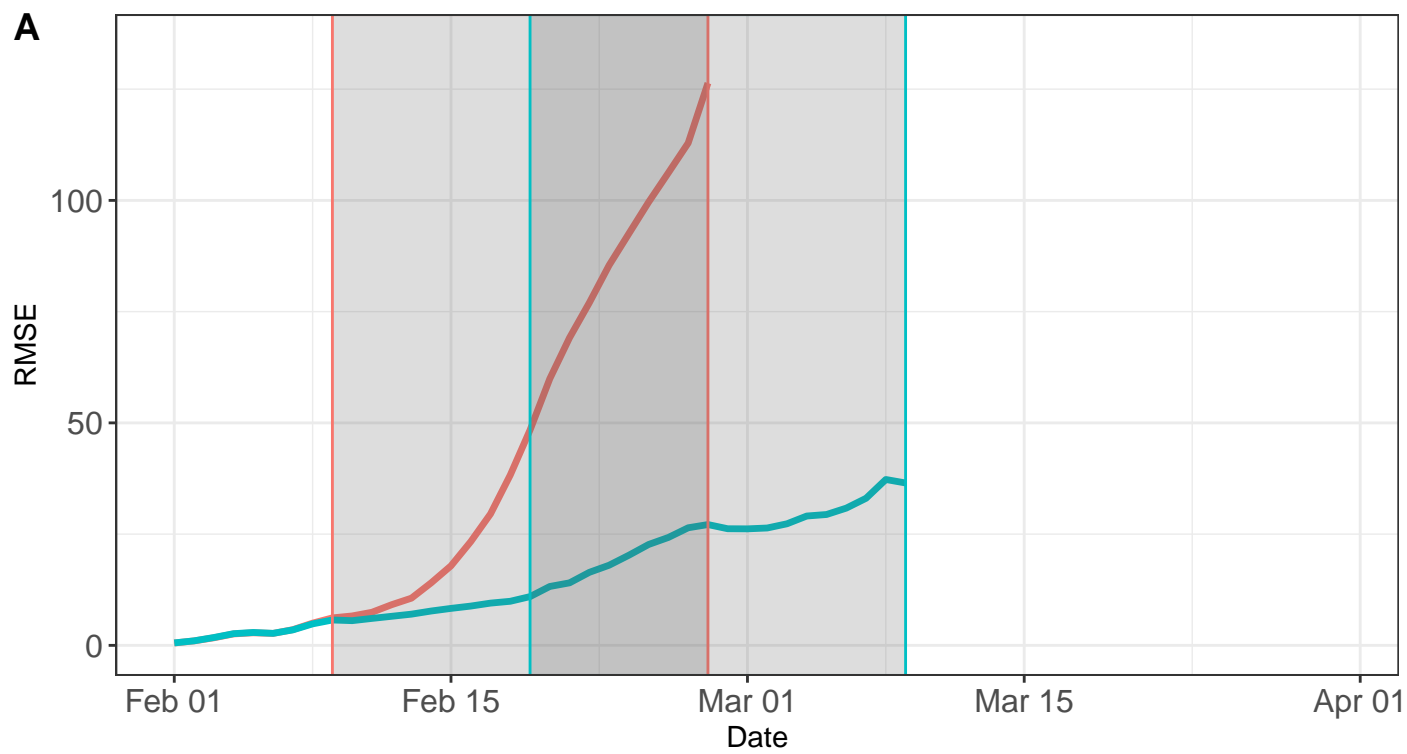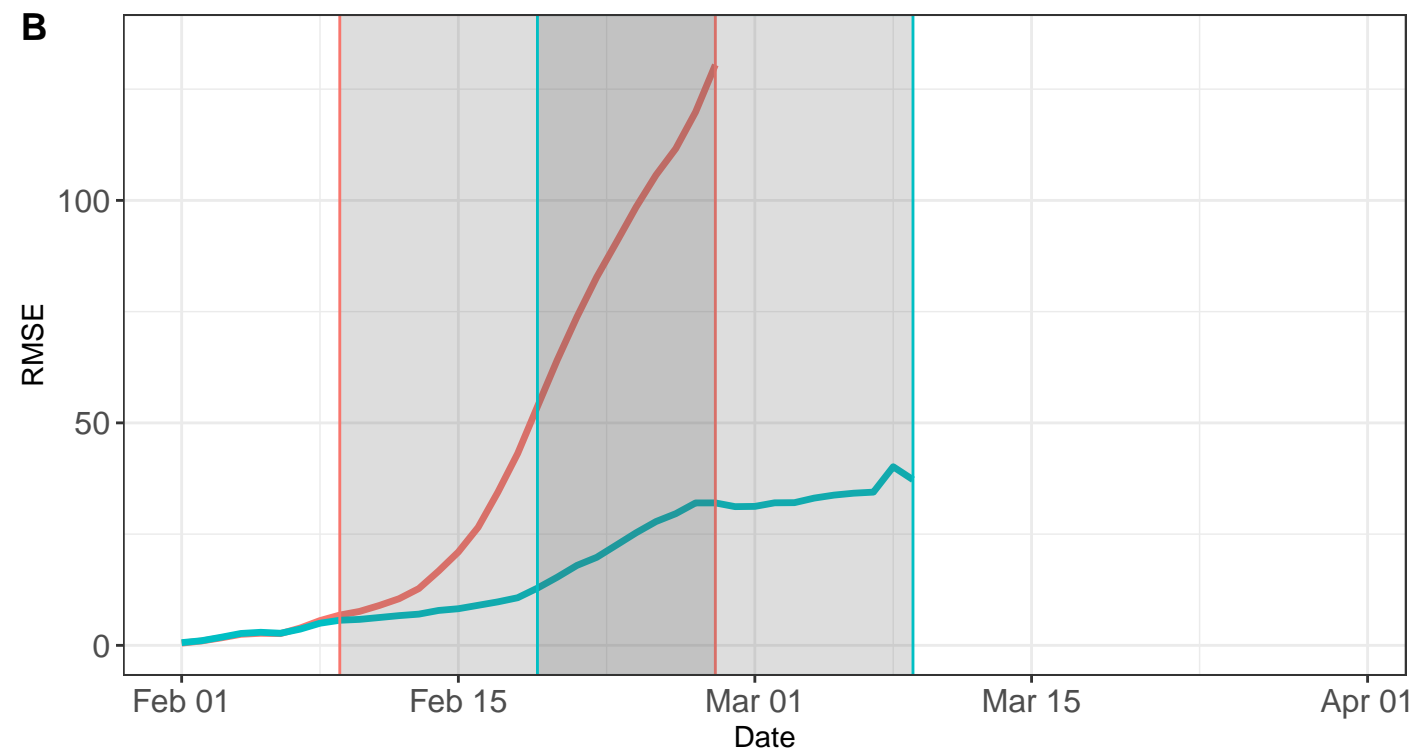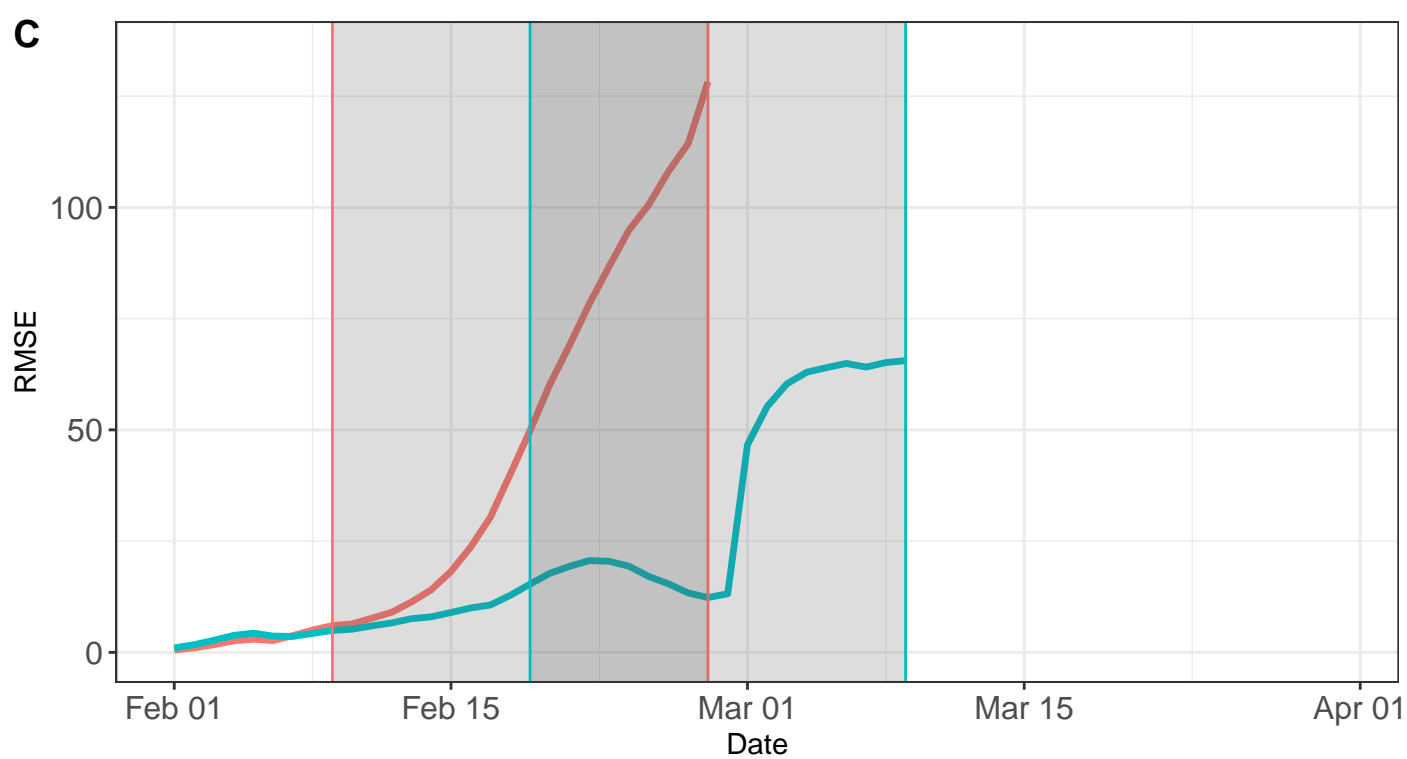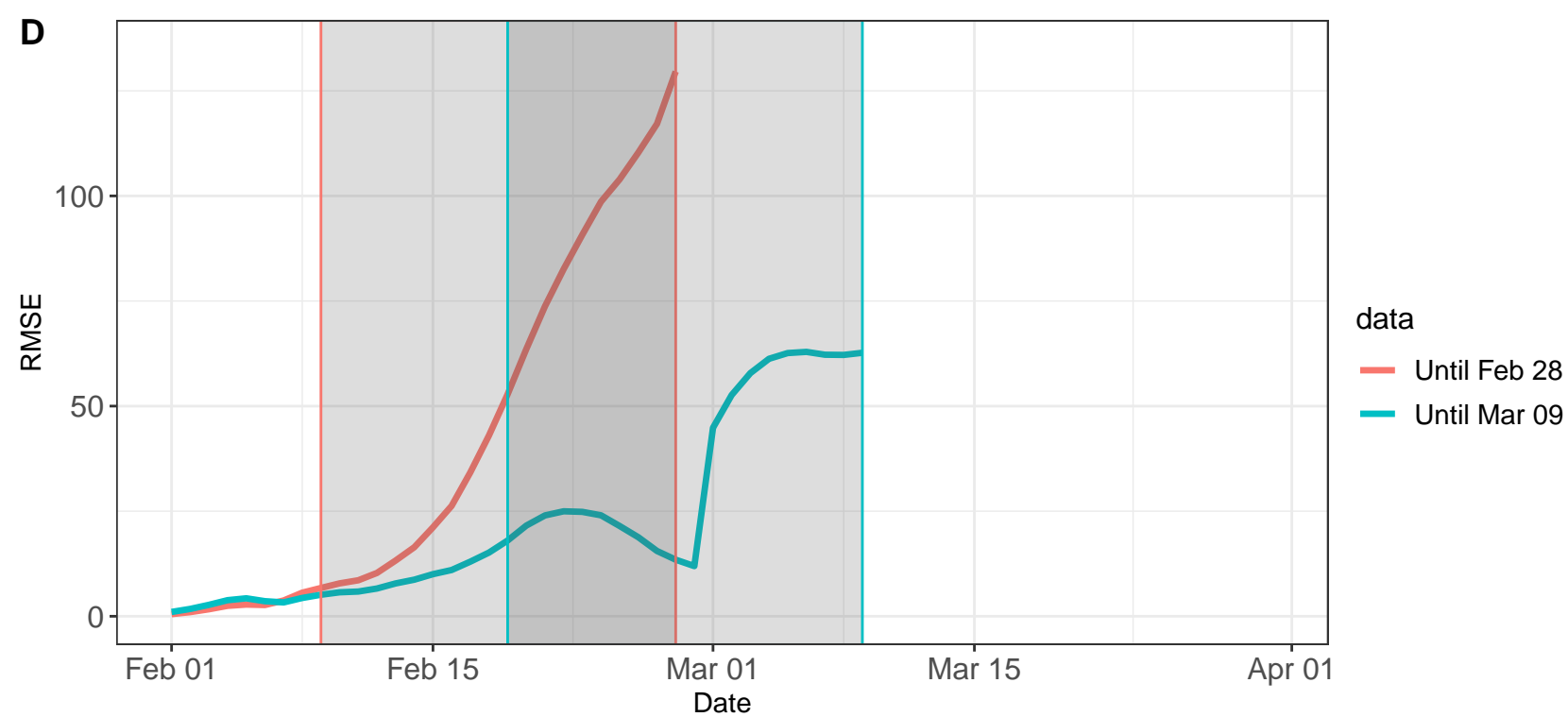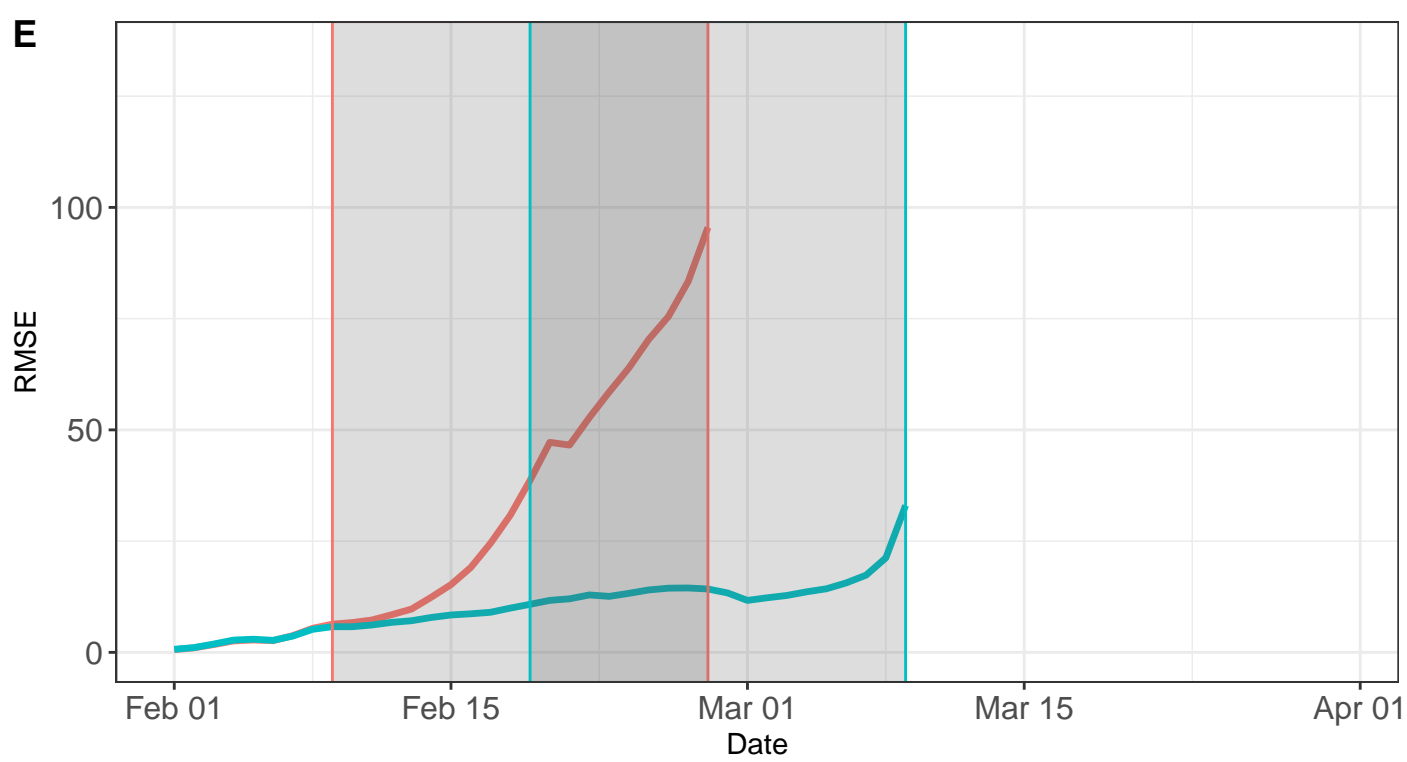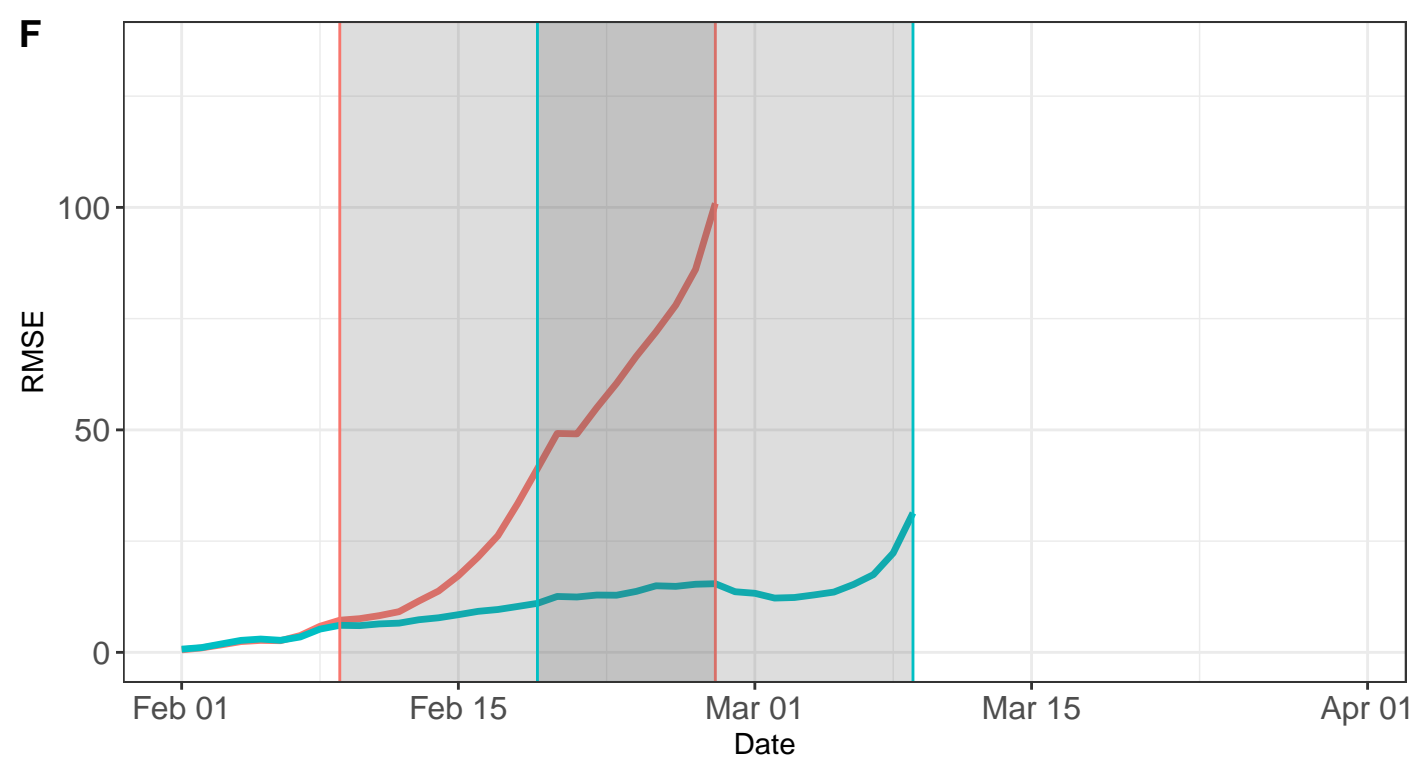

Supplement: S9 Fig — For all graphs: The colored curves represent different ending dates for line-list data, and their nowcasting regions are displayed as the gray-shaded areas with boundary lines in their corresponding colors. The RMSE were calculated based on 1000 simulated datasets. A: The RMSE given the reporting delay distribution was unchanged and l was correct. B: The RMSE given the reporting delay distribution was unchanged and l was incorrect. C: The RMSE given the reporting delay distribution was sharply improved and l was correct. D: The RMSE given the reporting delay distribution was sharply improved and l was incorrect. E: The RMSE given the reporting delay distribution was gradually improved and l was correct. F: The RMSE given the reporting delay distribution was gradually improved and l was incorrect. (PDF) [file pcbi.1009210.s009.pdf]
