# Supplementary material for: Bayesian back-calculation and nowcasting for line list data during the COVID-19 pandemic
Source: PLoS Comput Biol. 2021 Jul 12;17(7):e1009210. doi: 10.1371/journal.pcbi.1009210 (PMC8297945; doi:10.1371/journal.pcbi.1009210)
Supplement: S1 Text — (PDF) [file pcbi.1009210.s010.pdf]

Appendix: Bayesian back-calculation and nowcasting for line list data during the COVID-19 pandemic

Tenglong Li, Laura F. White

April 16, 2021

1 Simulation design

We simulated epidemics similar to Covid-19. We assumed the local epidemic started with 100 initial cases and do not allow for imported cases in the simulation. The time-varying reproductive number R_t was set based on literature [1, 2], to reflect the covid epidemic at multiple stages such as initial outbreak, lockdown as well as lift of lockdown (Fig 1). The distribution of generation interval was gamma distribution with shape and rate equal to 4.29 and 1.18, respectively, consistent with the literature [3]. The distribution of incubation period was lognormal distribution with mean and standard deviation of 1.621 and 0.418 respectively [4, 5]. We considered three factors in the simulation design, namely the reporting delay distribution, the maximum delay assumption and the data availability. In total, we had 18 different simulation scenarios ($3 \times 3 \times 2$).

The reporting delay distribution could be one of the following three scenarios:

1. No improvement: The reporting delay distribution was negative binomial distribution with $\mu = 9$ and $r = 3$ and it did not change.
2. Sharp improvement: The reporting delay distribution was negative binomial distribution with $\mu = 9$ and $r = 3$ before March 1, 2020 and

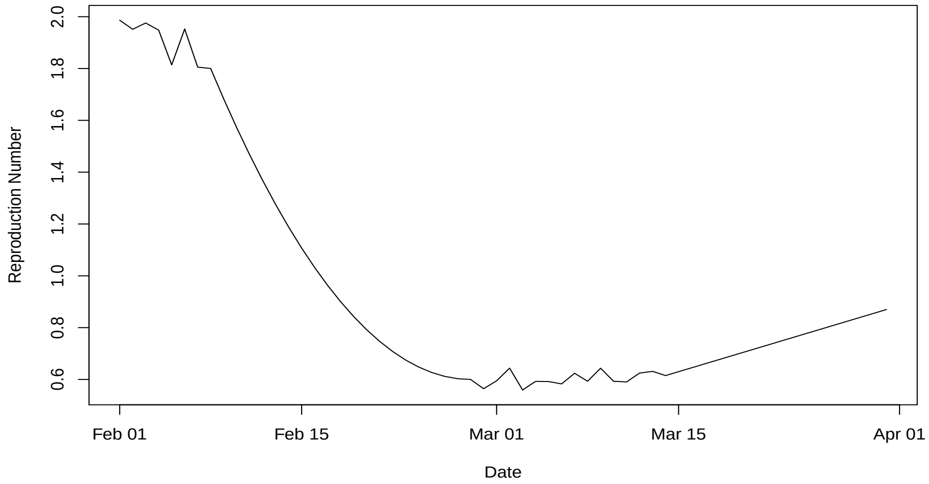

Figure 1: **The underlying time-varying reproductive numbers for the simulation.** This graph illustrates the time-varying reproductive numbers we used in the simulation. Notably, they corresponded to infection events from Feb 1, 2020 to March 31, 2020.

was negative binomial distribution with $\mu = 4$ and $r = 3$ after March 1, 2020.

3. Gradual improvement: The reporting delay distribution was negative binomial distribution with r fixed at 3. μ was set to 9 initially, then it got one day smaller every ten days from Feb 11, 2020 and finally $\mu = 4$ from March 22, 2020.

The maximum delay assumption could be one of the following two scenarios:

1. Correct maximum delay: l was assumed to be 20 for our Bayesian algorithm and it was actually 20 in the simulation.
2. Incorrect maximum delay: l was assumed to be 20 for our Bayesian algorithm and it was actually 25 in the simulation.

The data availability could be one of the following three scenarios:

1. Complete data: The line list data covered all cases reported from Feb 1, 2020 to March 31, 2020.

2. Delayed surveillance initiation: The line list data covered all cases reported from the starting date k to March 31, 2020. k could be Feb 11, Feb 21, March 2 or March 12.

3. Real time estimation: The line list data covered all cases reported from day 1 to day w. w could be either Feb 28 or March 9.

The simulation was done via branching process, i.e., we first drew the number of infectees from a Poisson distribution whose mean was defined by the number of infectors and corresponding reproductive number. We then obtained the infection dates, the symptom onset dates and the case reporting dates from the distributions of generation interval, incubation period and reporting delays respectively for those infectees. Finally, a line list data was created based on individual symptom onset dates and case reporting dates.

References

[1] Sam Abbott, Joel Hellewell, Robin N. Thompson, Katharine Sherratt, Hamish P. Gibbs, Nikos I. Bosse, James D. Munday, Sophie Meakin, Emma L. Doughty, June Young Chun, Yung-Wai Desmond Chan, Flavio Finger, Paul Campbell, Akira Endo, Carl A. B. Pearson, Amy Gimma, Tim Russell, Stefan Flasche, Adam J. Kucharski, Rosalind M. Eggo, and Sebastian Funk. Estimating the time-varying reproduction number of SARS-CoV-2 using national and subnational case counts. *Wellcome Open Research*, 5:112, 2020.

[2] Chong You, Yuhao Deng, Wenjie Hu, Jiarui Sun, Qiushi Lin, Feng Zhou, Cheng Heng Pang, Yuan Zhang, Zhengchao Chen, and Xiao-Hua Zhou. Estimation of the time-varying reproduction number of covid-19 outbreak in china. *International Journal of Hygiene and Environmental Health*, page 113555, 2020.

[3] Tapiwa Ganyani, Cécile Kremer, Dongxuan Chen, Andrea Torneri, Christel Faes, Jacco Wallinga, and Niel Hens. Authors' response: Estimating the generation interval for COVID-19 based on symptom onset data. *Eurosurveillance*, 25(29):18–19, 2020.

[4] Stephen A. Lauer, Kyra H. Grantz, Qifang Bi, Forrest K. Jones, Qulu Zheng, Hannah R. Meredith, Andrew S. Azman, Nicholas G. Reich, and Justin Lessler. The incubation period of coronavirus disease 2019 (CoVID-19) from publicly reported confirmed cases: Estimation and application. *Annals of Internal Medicine*, 172(9):577–582, 2020.

[5] Natalie Linton, Tetsuro Kobayashi, Yichi Yang, Katsuma Hayashi, Andrei Akhmetzhanov, Sung-mok Jung, Baoyin Yuan, Ryo Kinoshita, and Hiroshi Nishiura. Incubation Period and Other Epidemiological Characteristics of 2019 Novel Coronavirus Infections with Right Truncation: A Statistical Analysis of Publicly Available Case Data. *Journal of Clinical Medicine*, 9(2):538, 2020.
